# The effect of the creative problem-solving approach on creative thinking skills

**DOI:** 10.3389/fpsyg.2026.1734855

**Published:** 2026-01-21

**Authors:** Esen Ersoy, Deniz Özcan Kara

**Affiliations:** 1 Department of Mathematics Education, Faculty of Education, Ondokuz Mayıs University, Samsun, Türkiye; 2 Department of Special Education, Faculty of Education, Ondokuz Mayıs University, Samsun, Türkiye

**Keywords:** creative problem-solving approach (CPS), creative thinking skills, Torrance tests of creative thinking (TTCT), fluency–flexibility–originality, mathematics education

## Abstract

This study aims to investigate the impact of the Creative Problem-Solving (CPS)model on students’ creative thinking skills in mathematics education. Recent studies have shown that despite the emphasis on creativity and higher-order thinking skills in mathematics curricula, many students still struggle to generate original ideas and flexible solution strategies, highlighting the need for instructional approaches that effectively support creative thinking. The research method employed is a case study, a type of qualitative research design. The collected data were analyzed through descriptive and content analysis. The study group consisted of five graduate students enrolled in the course “Creativity and Creative Problem Solving in Mathematics Teaching” at a state university located in the Black Sea Region during the 2024–2025 academic year. Two creative problem tasks—Ideas in a Box Handout and Trick or Treat—were used as data collection tools. In addition, to determine the effect of the CPS-based mathematics course on creative thinking, Torrance Tests of Creative Thinking (Verbal Forms A and B) were administered. The data were analyzed using content and descriptive analysis techniques. The results indicated that the creative thinking skills of students who participated in mathematics instruction based on the CPS model improved by the end of the process. The findings revealed that the CPS model made a positive contribution to students’ creative thinking in mathematics education. It can be concluded that implementing the CPS model in mathematics teaching may enhance students’ higher-order thinking skills.

## Introduction

Mathematics is not merely a field confined to symbols and numerical operations; it is also a discipline that develops individuals’ reasoning, analytical, and higher-order thinking skills (Schoenfeld, 2016). Mathematics education shapes students’ ways of thinking and enables them to evaluate problem situations from different perspectives (Schoenfeld, 1992). Therefore, problem solving is a cognitive process that lies at the heart of mathematics education [Polya, 1945; National Council of Teachers of Mathematics (NCTM), 2000]. Problem solving not only involves reaching the correct result but also includes discovering solution paths, developing strategies, and structuring the solution process (Lester, 2013). It allows students to make sense of mathematical concepts, establish conceptual connections, and link abstract thinking with concrete situations (Schoenfeld, 1992). Effective problem-solving processes require students to think not only in terms of procedural knowledge but also in terms of conceptual understanding (Jonassen, 2000). For this reason, contemporary mathematics education considers problem solving not merely as an instructional tool but also as a component of creative thinking. From this perspective, problem-solving is viewed as a process in which students generate multiple solution paths, make non-routine connections between mathematical ideas, and reformulate given situations in novel ways. Rather than applying only known algorithms, students are encouraged to pose new problems, explore alternative strategies, and justify their reasoning, all of which are central to creative thinking. Thus, engaging in mathematical problem-solving provides a natural context for developing creativity-related skills, such as fluency, flexibility, and originality.

Mathematics education in the 21st century is increasingly viewed not only as a means of developing procedural fluency and conceptual understanding but also as a domain for nurturing higher-order thinking, creativity, and problem-solving skills (OECD, 2024; Schoenfeld, 2016). The global shift toward knowledge-based and innovation-driven economies has reinforced the importance of fostering learners’ creative and analytical capacities to address novel and complex problems (Leikin and Lev, 2013). Mathematics, as a discipline, inherently provides opportunities for developing these competencies by encouraging students to reason abstractly, explore multiple representations, and construct logical arguments [National Council of Teachers of Mathematics (NCTM), 2000; Polya, 1945].

Recent research emphasizes that effective mathematical problem-solving extends beyond algorithmic reasoning and involves the creative restructuring of knowledge, the generation of new solution paths, and flexible cognitive processing (Silver and Cai, 2005). This expanded view of mathematical proficiency aligns with Kilpatrick et al.’s (2001) framework, which situates adaptive reasoning and strategic competence as integral dimensions of mathematical thinking. Creative thinking is the process through which individuals reorganize their existing knowledge to generate new ideas, form different connections, and develop solutions through alternative pathways (Guilford, 1967; Torrance, 1974). In the educational context, creativity is defined as the individual’s capacity to produce original ideas and transform these ideas into practical applications (Runco and Jaeger, 2012).

Problem-solving plays a central role in mathematics learning because it enables students to actively construct mathematical meaning rather than passively receive information. Through problem solving, learners engage with mathematical concepts, identify relationships, and apply reasoning strategies that deepen their conceptual understanding. The process enables students to connect abstract ideas with real-world situations, promotes flexible thinking, and fosters the development of higher-order cognitive skills. In this sense, mathematics learning and problem-solving are mutually reinforcing: mathematics provides the structure and tools needed for solving problems, while problem-solving offers the context in which mathematical knowledge becomes meaningful and applicable. Therefore, integrating problem-solving into mathematics instruction is considered essential for fostering conceptual understanding, procedural fluency, and creative thinking.

The mathematics learning outcomes examined in this study reflect competencies emphasized at the graduate level, particularly in advanced teacher education programs. These outcomes include the ability to analyze mathematical situations, generate multiple solution strategies, justify reasoning processes, and transform mathematical problems into new forms—skills closely aligned with the dimensions of creative thinking such as fluency, flexibility, and originality. Additionally, students are expected to integrate theoretical perspectives on creativity and problem-solving into instructional decision-making, demonstrating both pedagogical and mathematical proficiency. Thus, the learning outcomes at this level extend beyond content mastery to encompass the capacity to design, evaluate, and implement problem-solving tasks that nurture creativity in mathematics education.

Problem-solving is essential in mathematics learning because it transforms mathematics from a set of procedures into an active, inquiry-based discipline. Through problem solving, students engage in reasoning, decision-making, and reflection, all of which support deeper conceptual understanding. Moreover, problem-solving encourages learners to move beyond routine exercises and explore non-standard problems that require creativity, persistence, and flexible thinking. These skills are not only fundamental to mathematical proficiency but are also aligned with 21st-century competencies. In contemporary mathematics education, problem solving is viewed as both a learning goal and a process through which all other mathematical competencies—such as communication, reasoning, representation, and connection—are developed. As such, problem-solving functions as a bridge between mathematical knowledge and innovative thinking.

Creative ideas are essential for students in mathematics learning because they enable learners to move beyond memorization and procedural execution toward flexible and innovative reasoning. Creativity in mathematics supports the ability to generate multiple solution pathways, reinterpret problems from different perspectives, and establish novel connections between mathematical concepts. These capacities are critical for dealing with non-routine problems, which more accurately characterize real mathematical thinking than routine exercises. Moreover, creative thinking fosters perseverance, curiosity, and intellectual autonomy—skills that not only contribute to mathematical success but also align with broader educational goals in the 21st century. When students develop creative ideas, they become active constructors of mathematical meaning, capable of transferring their understanding to new and complex situations. For this reason, creativity is increasingly recognized as a central component of mathematical proficiency and a foundational element of problem-solving-oriented instruction.

The creativity levels of mathematics students vary widely and are influenced by instructional practices, prior experiences, and opportunities to engage in open-ended problem solving. While mathematics is sometimes perceived as a discipline with fixed answers, research indicates that students can demonstrate high levels of creativity when they are encouraged to explore alternative methods, pose their own problems, and justify their reasoning. In particular, creativity in mathematics is evident in students’ fluency—producing numerous ideas; flexibility—shifting between different strategies; and originality—generating uncommon or unique solutions. Studies show that mathematics students often possess strong analytical abilities, but their creativity becomes more evident when learning environments adopt approaches such as Creative Problem Solving (CPS), inquiry-based learning, and rich problem contexts. Therefore, students’ creativity is not a fixed trait but a skill that can be nurtured through intentional instructional design, reflective activities, and opportunities for divergent thinking.

Creativity has been increasingly recognized as a core competence for lifelong learning (UNESCO, 2023). In mathematics education, it fosters curiosity, cognitive flexibility, and openness to ambiguity—skills vital for addressing real-world challenges (Cropley, 2019; Liljedahl, 2021). Creative thinking allows learners to explore unconventional patterns, reframe problems, and integrate knowledge across contexts (Runco and Jaeger, 2012; Treffinger and Isaksen, 2023).

Although creativity and problem-solving have become prominent competencies in contemporary mathematics education, the literature still shows a limited understanding of how structured approaches—such as the Creative Problem-Solving (CPS) approach—directly influence students’ creative thinking processes. While previous studies, including those by Mumford et al. (1991, 1996, 2012) have explored the stages and cognitive mechanisms of CPS, there remains a gap regarding how CPS-based instructional practices function specifically within mathematics learning environments and how they affect creativity components such as fluency, flexibility, and originality. This study addresses this gap by examining the impact of a CPS-based instructional process on graduate students’ creative thinking skills through both creative problem tasks and the Torrance Tests of Creative Thinking (Verbal A and B). By integrating CPS theory with practical mathematics tasks, the study offers a novel contribution to the field, highlighting not only the applicability of CPS in mathematics education but also demonstrating how CPS contributes to measurable improvements in creative thinking.

Creativity and creative problem solving have been examined across a wide range of age groups, from early childhood to undergraduate education (Scott et al., 2024; VanGundy, 2005; Stevenson et al., 2014; Sali, 2015; Kleibeuker et al., 2016; Hoffmann and Hills, 2021; Albar and Southcott, 2021; Smare and Elfatihi, 2024). Creativity and creative problem-solving are cognitive abilities that are included among educational objectives and assessed at different developmental levels. When the developmental stages of individuals are examined, the development of creativity and creative problem-solving skills among adult groups also becomes a significant area of interest.

Creativity theorists have long advocated for integrating creativity instruction at all levels of education and training (Kasyfi, 2021; Griffiths, 2014; Shaheen, 2010; Brundrett, 2007). In the knowledge-based and rapidly changing world of the 21st century, possessing knowledge alone is no longer sufficient. The importance of creativity in acquiring and utilizing knowledge is indisputable (Runco and Jaeger, 2012; Sternberg, 2006; Beghetto and Kaufman, 2022).

Creativity stands out as a fundamental skill that should be placed at the core of education. It enables individuals to develop original ideas, find unconventional solutions to problems, and approach situations from diverse perspectives. Accordingly, educational systems should not only provide academic knowledge but also create learning environments that nurture and support creative potential as they prepare individuals for the future.

In mathematics, creative thinking contributes to students’ ability to develop multiple perspectives, discover new forms of representation, and construct flexible thinking strategies (Sriraman, 2005). Fostering mathematical creativity supports students in experimenting with different strategies and developing innovative approaches during the problem-solving process (Leikin and Lev, 2013). Creativity in mathematics involves an unconventional process that requires generating solutions that are difficult to grasp through a new perspective. For the answers sought in problems, it is important to reveal higher-level knowledge and diverse viewpoints. Creative problems are those that do not have a single correct answer, allow for multiple solution paths, and challenge students’ cognitive flexibility (Silver, 1997; Posamentier and Krulik, 2020). Such problems encourage students to use both analytical and intuitive thinking simultaneously.

Creative problem-solving is not merely the application of existing knowledge, but a holistic process that involves redefining the problem, generating alternative ideas, and evaluating these ideas (Mumford et al., 2012). This process represents a fundamental mode of thinking that enables individuals to produce innovative solutions to complex situations.

The Creative Problem Solving (CPS) model, originally introduced by Osborn (1953) and later refined by Isaksen and Treffinger (2004), has evolved into a comprehensive framework guiding creativity-based education. Recent empirical studies highlight CPS as an effective pedagogical model for promoting metacognitive regulation, collaborative reasoning, and originality across STEM disciplines (Scott et al., 2009; Smare and Elfatihi, 2024). The cyclical structure of CPS—comprising stages of problem identification, idea generation, solution development, and implementation—encourages learners to iterate between divergent and convergent thinking, leading to innovative and feasible solutions (Treffinger and Isaksen, 2023). In mathematics education, CPS-based instruction facilitates a deeper engagement with problem situations, enabling students to approach abstract concepts through creative exploration rather than rote procedures (Bolden et al., 2010; Leikin and Lev, 2013).

The integration of CPS into mathematics teaching has gained renewed attention in the post-pandemic era, as digital and hybrid learning environments demand new forms of creativity and adaptability (OECD, 2024).

In mathematics education, creativity is grounded in knowledge. It involves breaking away from established ways of thinking, considering new possibilities, and applying a broad range of mathematical knowledge to construct something that did not exist before (Bolden et al., 2010). Within the context of mathematics education, CPS aims to enrich students’ cognitive flexibility and reasoning processes. In particular, the “adaptive reasoning” component emphasized in Kilpatrick et al.’s (2001) framework of mathematical proficiency—referring to justifying mathematical inferences, establishing logical relationships among results, and shifting between different representations or strategies—is explicitly supported by CPS.

The “ideate” and “develop” stages of CPS activate both inductive and deductive reasoning, allowing students to compare solution paths, discuss their rationales, and strengthen their arguments. Therefore, the creative problem-solving levels of graduate students in mathematics education—especially their performance in idea generation and development stages leading to a solution—are of great importance.

In this context, the present study investigates the impact of the CPS model on graduate students’ creative thinking skills within mathematics education. By employing Torrance’s (1974) framework and Mumford et al.’s (1991) CPS criteria, the study examines how participants construct, reorganize, and evaluate ideas during creative mathematical problem-solving tasks. The findings contribute to the growing body of literature emphasizing the transformative potential of CPS-oriented instruction in developing creative, flexible, and adaptive mathematical thinkers for the 21st century.

The main purpose of this study is to examine the effect of the Creative Problem Solving (CPS) model on students’ creative thinking skills in mathematics education. Within the scope of the research, the changes in the creative thinking levels of graduate mathematics teacher candidates resulting from their participation in the CPS process were identified. Accordingly, the study analyzed the extent to which students developed creative ideas during problem-solving processes, generated alternative solution paths, and demonstrated their original thinking skills. In light of the information mentioned above, answers were sought to the following three sub-problems.

### Sub-problems

What are the analysis results of the responses given by the study group to the creative problem titled “Ideas in a Box Handout” according to the creative problem-solving criteria?

What are the analysis results of the responses given by the study group to the creative problem titled “Trick or Treat” according to the creative problem-solving criteria?

How does the comparison of the study group’s responses to the two creative problems align with the scoring criteria of the creative thinking test?

## Methods

### Research design

This study is a qualitative research that aims to examine the effect of creative problem-solving practices in mathematics instruction on students’ levels of creativity. The case study design was adopted in the research. The case study was preferred because it allows an in-depth and detailed examination of a specific phenomenon or practice (Yıldırım and Şimşek, 2018). In this context, the study aims to reveal the reflections of creative problem-solving practices conducted in mathematics education courses on students’ creative thinking skills.

A case study design was adopted in this research because the aim of the study was to conduct an in-depth investigation of how graduate students experience and respond to the Creative Problem-Solving (CPS) process within the context of mathematics learning. Case studies are particularly suitable for examining complex phenomena that are strongly shaped by individual experiences, instructional interactions, and contextual factors. In this study, the CPS-based mathematics instruction constituted a unique case, as students had been engaging with mathematical problem solving and creativity-oriented tasks throughout the semester.

The case study approach allowed the researchers to explore the participants’ creative thinking processes, their responses to the CPS tasks, and the development of their fluency, flexibility, and originality in detail. Through multiple data sources, including creative problem tasks and the Torrance Tests of Creative Thinking, the case study provided a comprehensive understanding of how CPS instruction influences creative thinking. The strength of this design lies in its ability to capture the richness of the participants’ experiences rather than reducing them to numerical outcomes.

Furthermore, the case study concluded with the convergence of perspectives between the researchers and the participants, achieved through member checking and reflective discussions. This agreement strengthened the credibility and trustworthiness of the findings, ensuring that the interpretations accurately reflected the participants’ experiences.

Although the study includes pre–post administration of the Torrance Tests of Creative Thinking (Verbal Forms A and B), the primary aim of the research was not to examine causal effects or statistically compare pre–post scores. Instead, the study was designed as a qualitative case study to explore, in depth, how graduate students experienced and engaged with the Creative Problem-Solving (CPS) process over an extended instructional period.

The case under investigation represents a naturally occurring instructional context in which students participated in CPS-based mathematics instruction throughout the semester. The use of Torrance Verbal Forms before and after the implementation served a supportive and descriptive function, allowing the researchers to qualitatively examine changes in students’ creative thinking expressions rather than to test hypotheses or calculate effect sizes. Therefore, the Torrance data were analyzed through content analysis, focusing on the dimensions of fluency, flexibility, and originality, in alignment with the qualitative nature of the study.

No quantitative comparison (e.g., score differences or statistical tests) was conducted because the small sample size (*n* = 5) and the case study design do not support meaningful statistical inference. Moreover, Torrance tasks are multidimensional and yield overlapping indicators of creative thinking, making qualitative interpretation more appropriate for capturing the richness of participants’ responses. Accordingly, the combination of qualitative CPS task analysis and qualitative interpretation of Torrance responses is theoretically grounded and consistent with the exploratory purpose of the study.

### Study group

The study group of this research consists of a total of five students enrolled in the “Creativity and Creative Problem Solving in Mathematics Education” graduate course within the Mathematics Education Master’s Program at the Faculty of Education of a state university located in the Black Sea Region. The study group was determined through criterion sampling, a purposeful sampling method. The criterion set for selection was that the participants were enrolled in the “Creativity and Creative Problem Solving in Mathematics Education” course and volunteered to participate in the study. The ages of the participants ranged from 22 to 26. The study group consisted of three female and two male students.

In this study, a purposeful sampling technique was used to determine the participants. Purposeful sampling is widely preferred in qualitative research because it enables the selection of individuals who can provide rich, relevant, and in-depth information about the phenomenon under investigation. The sample was composed of graduate students enrolled in the “Creativity and Creative Problem Solving in Mathematics Teaching” course, as these participants had direct experience with the CPS-based instructional process implemented in the research.

The primary criterion for inclusion was the students’ active participation in the course activities involving creative problem tasks and CPS steps. Therefore, the sample was intentionally limited to five students—two male and three female—who were able to contribute meaningful insights into the development of creative thinking within a CPS-oriented mathematics learning environment. This selection strategy allowed the researchers to obtain detailed data that aligned with the aims of the case study design.

As this study was designed as a qualitative case study, the goal was not to obtain a sample that statistically represents a large population, but rather to gain an in-depth understanding of the participants’ creative thinking processes within a CPS-based instructional context. In qualitative research, especially in case studies, smaller samples are intentionally selected because they allow for detailed exploration of complex cognitive and instructional phenomena that cannot be meaningfully captured through large-scale sampling. Therefore, the inclusion of five graduate students is consistent with the methodological purpose of the study and supports the depth of analysis required for examining CPS processes. Regarding generalizability, qualitative case studies aim for analytical rather than statistical generalization. The purpose is not to generalize findings to all populations but to provide rich descriptions and insights that can inform similar educational contexts. The results contribute to theory-building by demonstrating how CPS-based mathematics instruction may influence creative thinking, and these insights may guide future studies or instructional designs in comparable settings.

### Data collection tools

During the course, data were collected through various creative problems. In this study, two creative problems—Ideas in a Box Handout and Trick or Treat—were included. These problems were structured to enable students to generate diverse ways of thinking, develop alternative solutions, and present original ideas. During the implementation phase of the creative problems, the students enrolled in the course worked together as a group. Information regarding the creative problems applied in the process is presented below in order.

### Creative Problem 1: Ideas in a box handout

The problem was adapted from VanGundy’s (2005) book “101 Activities for Teaching Creativity and Problem Solving.” The problem was translated into Turkish and reviewed by two language experts to ensure linguistic validity through expert opinions. Prior to the main implementation, the problem was administered to a pilot group to establish content validity.

Students were presented with the worksheet below. In the worksheet, they were asked to imagine themselves as design managers of a food company tasked with increasing the company’s declining sales. They were instructed to design and market the product in the best possible way, taking into account an eye-catching package design and unique features. The students were informed that they had complete freedom during the design phase.
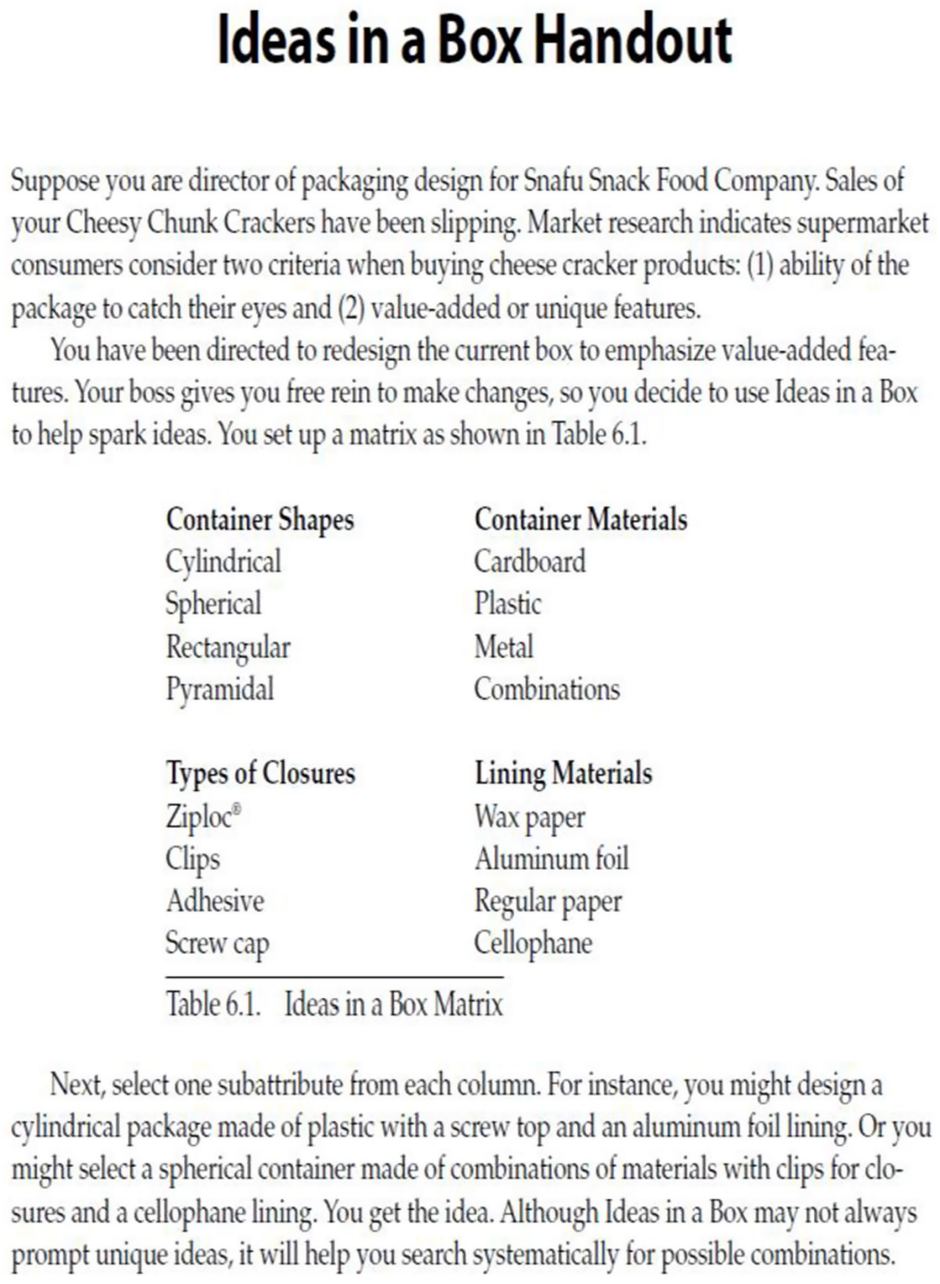


### Creative Problem 2: Trick or treat

The problem was adapted from Price’s (2006) book “Creative Maths Activities for Able Students: Ideas for Working with Children Aged 11 to 14.” The problem was translated into Turkish and reviewed by two language experts to ensure linguistic validity through expert opinions. Before the main implementation, the problem was administered to a pilot group to check content validity.

Students were presented with the worksheet below. The worksheet included a map of an area, where certain locations along the roads were numbered and represented as houses. Students were asked to examine the positions and distances of the houses on the map. It was stated that the distance between houses numbered 3 and 4 was 300 meters, and that this distance could be walked within 5 min. Each house was assumed to be inhabited, and greeting the residents took approximately 2 min. Each resident who was greeted would give 10 candies in return for the greeting. Based on this scenario, students were asked to start from house number 10, visit as many houses as they wished, and return to house number 10 within 3 h. They were expected to visit multiple houses within the 3-h period, collect as many candies as possible, and return home on time.

A minor revision was made before the problem’s implementation. Upon reviewing the data provided in the original problem, it was found that since one mile equals 1,609.344 meters, 0.3 miles = 0.3 × 1,609.344 = 482.80 meters. However, to prevent students from dealing with a non-rounded figure like 482.80 meters, the value was adjusted to 300 meters. This change was made to make the task more comprehensible, as the aim was not to measure mathematical computation skills.
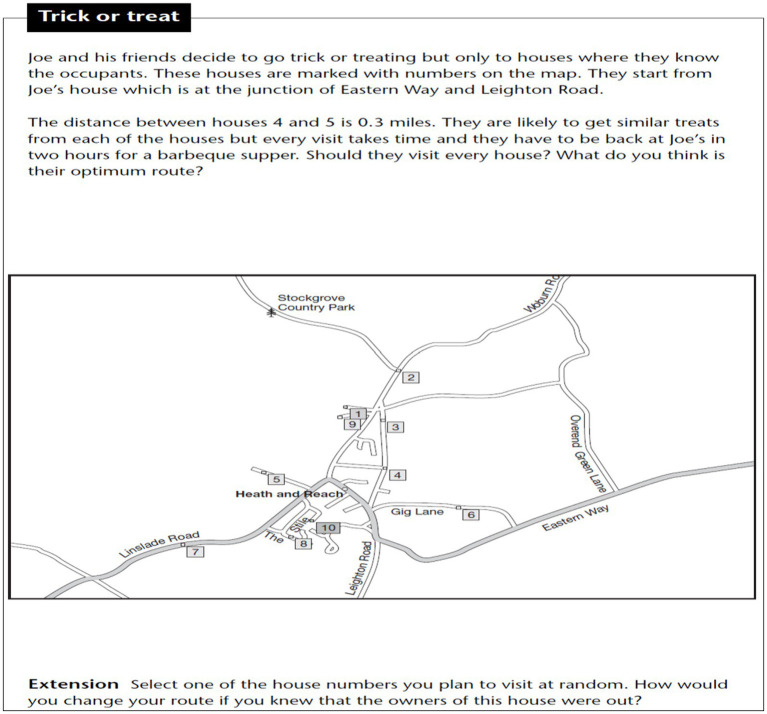


In this study, one of the criteria used was the Torrance Tests of Creative Thinking (TTCT), specifically the Verbal Forms A and B. This criterion was selected to determine the effect of the creative problem-solving course on creative thinking.

### Torrance tests of creative thinking (TTCT)—verbal forms A and B

This test measures creativity through verbal stimuli and assesses three dimensions: fluency, flexibility, and originality. In the research, Form A was administered before the implementation, and Form B was administered after the implementation. The Turkish adaptation of the test was developed by Aslan (2001), and its validity and reliability have been studied.

### Torrance tests of creative thinking

The Torrance Tests of Creative Thinking (TTCT) used in this research are suitable for the graduate students included in the study, as the test can be administered from kindergarten through adulthood. The test consists of two subtests—verbal and figural—and includes a total of 10 items (Aslan, 2001). In the TTCT, the tasks do not correspond to single dimensions; rather, each task simultaneously elicits indicators of fluency, flexibility, originality, and elaboration. Therefore, the items cannot be numerically categorized by creative thinking dimensions.

To measure creativity, one of the variables in the study, the Verbal Form of the Torrance Tests of Creative Thinking (TTCT) was employed. Developed by Torrance (1966), the test is composed of two main sections: verbal and figural.

The subtests in the verbal section include: asking questions, guessing causes, guessing consequences, product improvement, unusual uses, unusual questions, and “just suppose” activities (Aslan, 2001). In the first week of the course, the TTCT Verbal Form A was administered, and at the end of the semester, the TTCT Verbal Form B was used to determine the development of creative thinking.

Each subtest in the forms was evaluated across three dimensions: fluency, flexibility, and originality. Information about each of these dimensions is provided below.

Fluency, the first component, refers to the ability to generate numerous ideas within a given timeframe. This quantitative aspect of creativity reflects an individual’s capacity to produce relevant responses to a given problem (Kaufman and Sternberg, 2010). In the fluency dimension, generating a greater number of ideas also brings about more innovative solutions, as presenting a wide range of perspectives and ideas leads more quickly to creative outcomes. In this study, the ideas written by the participants were evaluated within the scope of the fluency dimension.

Flexibility represents the capacity to generate diverse categories of responses and approach problems from multiple perspectives. This component reflects cognitive adaptability and the ability to break free from mental sets or fixed thinking patterns. Studies have demonstrated that flexibility in thinking significantly contributes to problem-solving success, particularly in complex or novel situations that require innovative approaches (Čančer and Mulej, 2013). Flexibility requires the ability to approach problem situations from multiple perspectives. In the flexibility dimension, individuals move beyond mental set patterns and transition toward innovative approaches. In this study, responses that demonstrated diverse and multiple perspectives by breaking away from conventional thinking patterns were evaluated within the scope of the flexibility dimension.

Originality, the most commonly associated component with creativity, refers to the ability to produce unique, unusual, or novel ideas. This component is often evaluated based on the statistical rarity of responses within a given population (Runco, 2022). In the originality stage, individuals who can think independently and use their imagination effectively generate unique and unconventional solutions to the problems they encounter. Such individuals present different and original responses in their proposed solutions and approaches.

### Research process

The research was conducted in three stages. In the first stage, the Torrance Tests of Creative Thinking—Verbal Form A were administered to the students before the process began. The students were given 35 min to complete the form.

In the second stage, creative problem-solving–oriented activities were implemented during the course over 14 weeks (3 h per week). For the application of the creative problems, a total of two class hours was allocated. During the first 4 weeks, the lessons were conducted theoretically, focusing on topics such as creativity, creative thinking, and creative problem-solving in mathematics education, in line with the curriculum. The students taking the course conducted literature reviews and participated in in-class evaluations during the lesson process. After the fourth week, the course transitioned to the practical implementation of creative problem-solving activities in mathematics education. The students worked collaboratively as a group to find solutions to all problems.

In the third stage, at the end of the semester, the Torrance Tests of Creative Thinking—Verbal Form B were administered. The students were again given 35 min to complete the form.

### Data analysis

The creative problems were evaluated through descriptive analysis, while the Torrance Verbal Forms were analyzed using content analysis. Descriptive analysis is an approach that enables data to be organized systematically and presented clearly by the researcher (Miles et al., 2014). In this study, the data were presented in an organized and explanatory manner, incorporating descriptions directly based on participants’ statements. In analyzing the solutions to the creative problems, student quotations were included, along with detailed descriptions and interpretations of all data.

As the study was designed as a qualitative case study, the data analysis was intentionally limited to content and descriptive analysis rather than inferential statistical procedures. Although the Torrance Tests of Creative Thinking yield numerical scores, these scores were not used for statistical comparison or hypothesis testing in this study. The decision not to conduct quantitative analyses (e.g., mean scores, standard deviations, or gain scores) was primarily based on the small sample size (n = 5) and the exploratory nature of the research, which do not support meaningful statistical inference. Instead, Torrance responses were analyzed qualitatively to capture changes in students’ fluency, flexibility, and originality through rich, context-sensitive interpretations. Since no numerical statistical analysis was performed, consultation with a statistician was not required. Maintaining a purely qualitative analytical approach allowed the researchers to preserve the depth and complexity of participants’ creative thinking processes, which aligns with the methodological framework and aims of the study.

The creative problems were analyzed according to the problem-solving criteria developed by Mumford et al. (1991). The coding procedure for these criteria is explained below: At the problem construction (1) stage, responses were scored based on types of reflection that demonstrated constraints, information, approaches, original goals, or high-quality reasoning. At the information encoding (2) stage, the steps developed by Mumford et al. (1996) were taken into consideration. In evaluating student responses, factors such as the time spent encoding information related to reality, instability, goals, constraints, as well as the value of information based on differences, relationships, and principles, were considered for scoring. The category search (3) and category selection (4) stages were assessed in accordance with the evaluation steps proposed by Mumford et al. (1996). These were evaluated based on specific action plans, general principles, and long-term goals. The category combination (5) and reorganization (6) stages were assessed using categorical examples developed by Mobley et al. (1992). The idea evaluation (7) and solution implementation and monitoring (8) stages were analyzed based on the evaluation criteria established by Mumford et al. (1991). In the overall evaluation, the total scores derived from these stages were used to assess the application of examples developed for the category combination and reorganization skills. Examples of evaluations to be considered in the analysis of student quotations, according to these criteria, are presented below.

**Table:** 

Criteria	Excerpts from Student Evaluations
Problem Construction	The problem must be correctly understood and rephrased by the student in their own words.
Information Encoding	Known information is identified. It is important to mentally encode and effectively use accurate information.
Category Search	Categories for different solution paths are explored. It is essential for the student to recognize possible categories of solutions.
Category Selection	The correct category is chosen, and the method of solution is determined.
Category Combination and Reorganization	The selected categories are combined, or a different line of reasoning is developed.
Idea Evaluation	The validity of the developed ideas is questioned. The selection among alternatives and the reasoning behind it are examined here.
Solution Implementation and Monitoring	The solution is implemented, the result is checked, and the accuracy of the solution is tested.

Content analysis is one of the fundamental analytical methods used in qualitative research to systematically, objectively, and meaningfully examine data. Berelson (1952) as cited in Tavşancıl and Aslan (2001) defined content analysis as a research technique that provides objective, systematic, and quantitative descriptions of the manifest content of communication. This technique involves coding the meaningful units found in participants’ statements, documents, or observation notes and grouping these codes under categories and themes. The aim is to reveal the implicit meanings, patterns, and relationships within the collected data (Krippendorff, 2018; Miles et al., 2014).

The scores obtained from the Torrance Verbal Test were evaluated under the main theme of “Creativity,” across the categories of “Asking Questions, Guessing Causes, Guessing Consequences, Product Improvement, Unusual Uses, Unusual Questions, and Just Suppose.” The changes in students’ levels of creativity were then interpreted from a qualitative perspective.

In this study, content analysis was conducted in four stages:

(1) Coding of Data:

The responses provided by the students in the Torrance Verbal Creative Thinking Test Forms A and B were examined, and meaningful expressions were converted into codes.

(2) Identification of Themes:

The coded data were grouped together, and main themes were established. As a result of the data analysis, under the theme of “Creativity,” the following categories were identified: Asking Questions, Guessing Causes, Guessing Consequences, Product Improvement, Unusual Uses, Unusual Questions, and Just Suppose.

(3) Organization and Interpretation of Data:

For each theme and category, students’ pre-test (Form A) and post-test (Form B) responses were compared, and changes observed during the implementation process were identified. For example, students who produced only one type of solution in the pre-test were observed to develop multiple solution methods after the implementation.

(4) Presentation of Findings:

The findings were supported with tables and direct quotations. In this study, the Torrance Verbal Forms A and B were evaluated considering the categories of Asking Questions, Guessing Causes, Guessing Consequences, Product Improvement, Unusual Uses, Unusual Questions, and Just Suppose.

### Validity and reliability in the research

Various strategies were employed to ensure the validity and reliability of the study. The Torrance Verbal Forms A and B, students’ responses, and answers to the creative problems were evaluated together. Thus, by using multiple data sources and data collection tools—rather than relying on a single source, method, or perspective—the accuracy and consistency of the research were tested, ensuring the triangulation strategy.

Before implementing the creative problems, expert opinions were obtained to establish content validity. In addition, some of the findings were shared with the students and verified through comparison with their views, thereby ensuring participant validation (member checking).

The findings obtained from the study were reported in detail to allow other researchers to review and evaluate them, providing a thick description of the process and results. Furthermore, two researchers independently evaluated the data, and a consensus was reached on the findings. As a result of the analyses conducted by two researchers, the inter-rater agreement was calculated as 86%.

### Findings

The findings related to each sub-problem are presented below in order.

What are the analysis results of the responses given by the study group to the creative problem titled “Ideas in a Box Handout” according to the creative problem-solving criteria?

What are the analysis results of the responses given by the study group to the creative problem titled “Trick or Treat” according to the creative problem-solving criteria?

When the responses given to both creative problems are compared with the scoring criteria of the creative thinking test, what are the results for the study group?

### Findings related to the first problem

The analysis results of the responses given by the study group to the creative problem titled “Ideas in a Box Handout” were evaluated according to the creative problem-solving criteria proposed by Mumford et al. (1991), as presented below.

#### Problem construction

**Figure 1:**
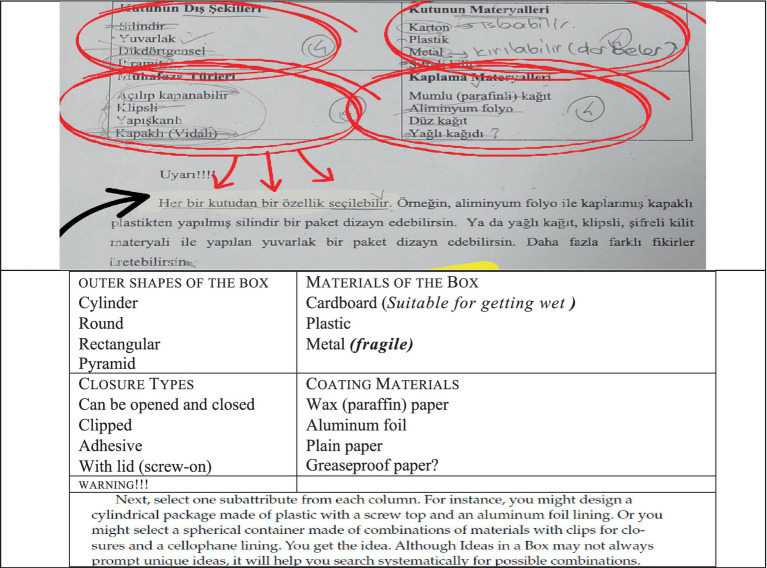
Solution figure for problem construction.

At this stage, brainstorming was conducted by reading and discussing the given elements of the problem. For example, when considering the material of the box, the possibility that a cardboard box might get wet and become unusable was initially mentioned. It was also noted that if the box were made of metal, it could be damaged or dented when exposed to impact (see Figure 1).

The original version of the student paper is shown in the first image above. The image below presents the English translation of the original text. The visual above shows that the students emphasized selecting features from each box and marked different characteristics for each one. In the problem construction stage, it can be concluded that the problem was correctly understood and that possible situations were noted.

#### Information encoding

**Figure 2:**
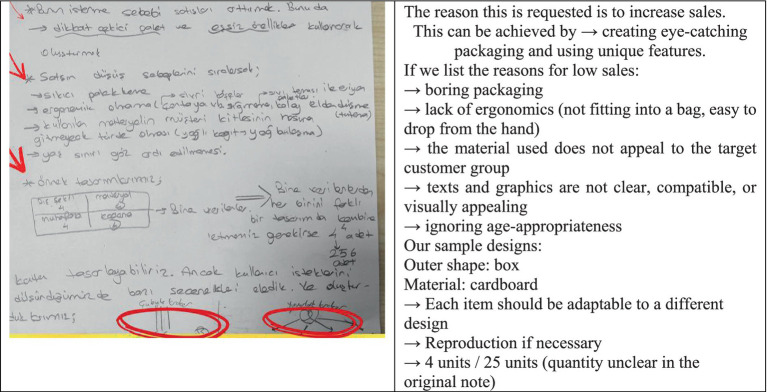
Solution figure for information encoding.

In the information encoding stage, it is essential to identify the known information. At this stage, while designing the box, the problem was approached from various perspectives—such as ensuring the design was economical, environmentally friendly, suitable for children’s health, and even considering the shape of the crackers when selecting the box type (see Figure 2).

The original version of the student paper is shown in the first image above, and the English translation of the original text is presented alongside it. According to the screenshot above, in the information encoding stage, students demonstrated different perspectives by designing a package that featured distinctive and eye-catching characteristics related to sales performance. To increase sales, they focused on the possible reasons for the decline, such as dull packaging and a lack of ergonomic design (e.g., sharp corners, packages that do not fit into a bag, easily slipping from the hand, or dissolving when exposed to liquid). They also noted that the material used might not appeal to the target customers—for instance, greasy paper that transfers oil stains. From the given features in the visual, it was determined that there were 4^4^ = 256 possible combinations. However, some options were eliminated when considering user or designer preferences. Ultimately, it can be concluded that the known information was identified, effectively encoded, and mentally utilized.

#### Category search

**Figure 3:**
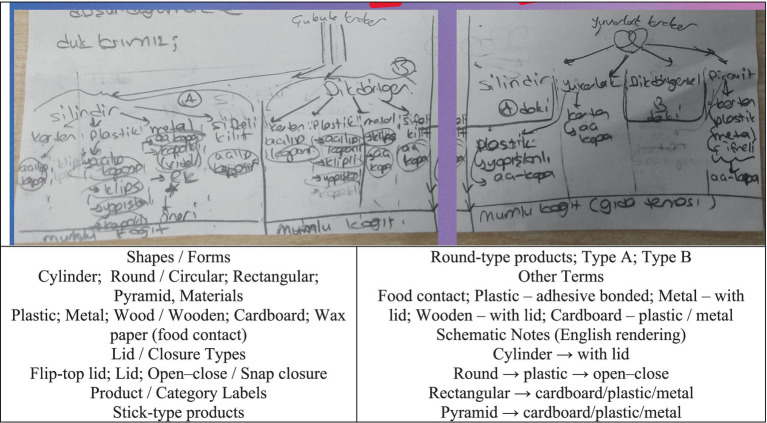
Solution figure for category search.

Within the allotted time for solving the problem, all possible solution paths were generated using diagrams and sketches. At this stage, it was considered that the stick crackers could take different shapes, such as cylindrical, rectangular, pyramidal, or circular (see Figure 3).

The original version of the student paper is shown in the first image above, and the English translation of the original text is presented alongside it. The visual above indicates that the outer shape of each box, the material of the box, the type of preservation, and the coating material were selected. Different alternatives were proposed for designs intended for both stick and round crackers. For stick crackers, a cylindrical shape was considered suitable, with options such as a cardboard container with a flip-top lid, a plastic container with a flip-top or clip-on lid, and a metal container with a screw-on lid. Additionally, suggestions included a coded lock and a flip-top cover for the cylindrical design. For rectangular-shaped stick cracker packages, a cardboard box with a flip-top lid was suggested; for plastic materials, flip-top, clip-on, or adhesive lids were considered; and for metal materials, clip-on or flip-top lids were proposed. Sensitivity to food contact was ensured by using waxed paper coating. In the round cracker design, four shape options (cylindrical, round, rectangular, and pyramidal) were again explored. For the round design, a flip-top lid was suggested, while for the rectangular design, the same approach as in the stick cracker packaging was adopted. For the pyramidal design, opinions were expressed about using cardboard, plastic, or metal materials with a coded flip-top lid. During the category search stage, categories for different solution paths were explored, and possible solution categories were successfully identified.

#### Category selection

**Figure 4:**
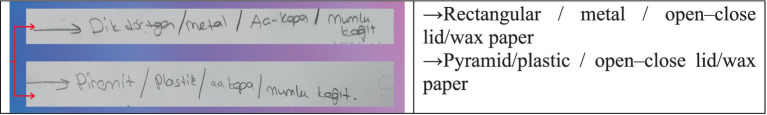
Solution figure for category selection.

Among all the possible solution paths identified, the most appropriate solutions to the problem are presented in the visual (see Figure 4).

The original version of the student paper is shown in the first image above, and the English translation of the original text is presented alongside it. The outer shape of the box was determined to be rectangular, made of metal material, designed with a flip-top feature, and coated with wax paper. For another box, the outer shape was chosen as a pyramid, the material as plastic, the preservation type as flip-top, and the coating as wax paper. As a result, the appropriate category was selected, and the method of solution was determined.

#### Category combination

**Figure 5:**
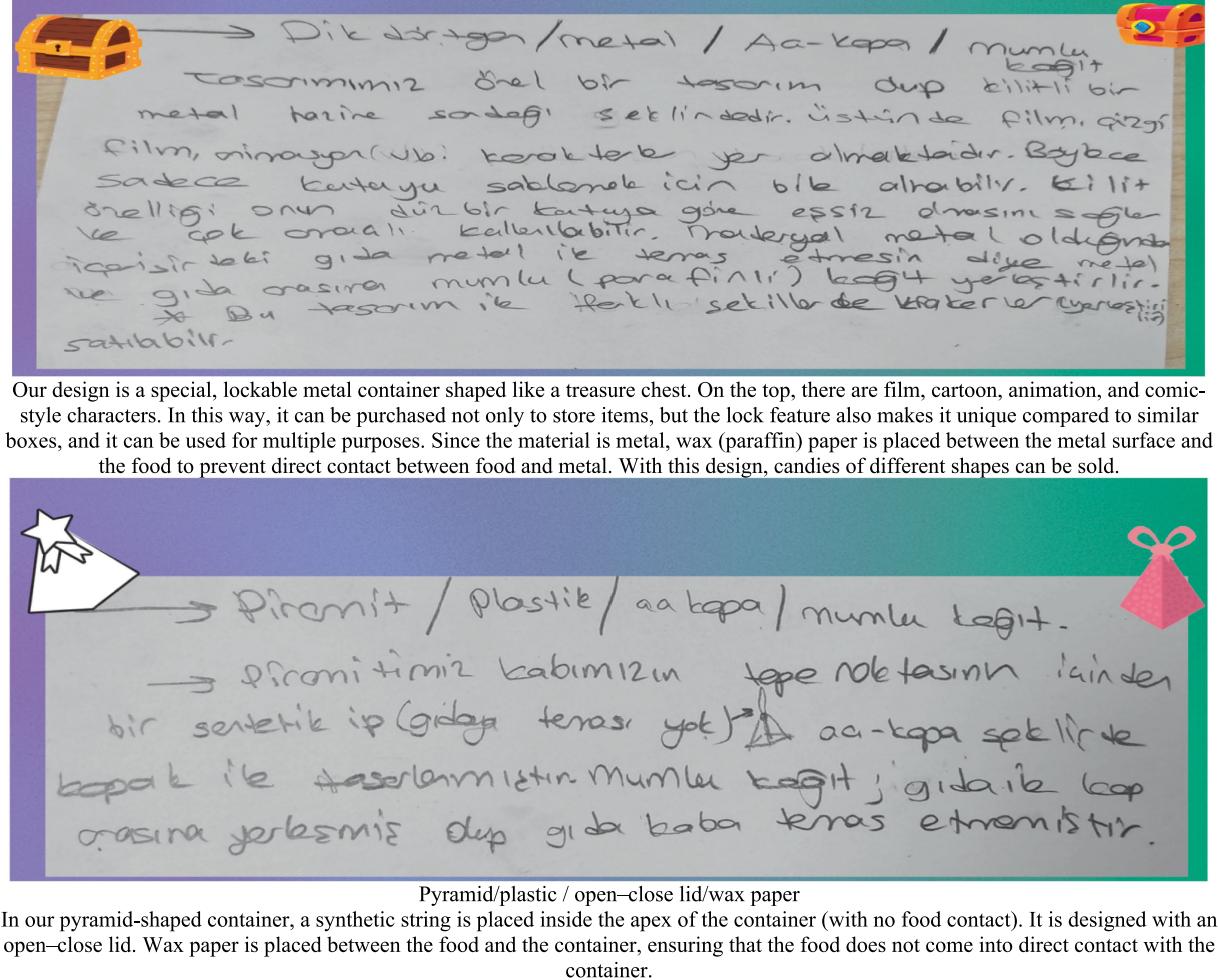
Solution figure for category combination.

The selections made to express the box design in a more concrete way are illustrated in the visual. Thus, the accuracy of the solution was also verified. The visual below indicates that the box’s outer shape could be rectangular, its material is metal, its preservation type is flip-top, and its coating material is wax paper (see Figure 5).

The original version of the student paper is shown in the first image above. The image below presents the English translation of the original text. At this stage, it was stated that the design would be a special one, created in the form of a locked metal treasure chest. The box shape itself would attract attention during the sales phase. The lock feature distinguishes it from an ordinary box. In addition, it was suggested that the interior be lined with wax paper to prevent the food from coming into direct contact with the metal.

#### Reorganization

**Figure 6:**
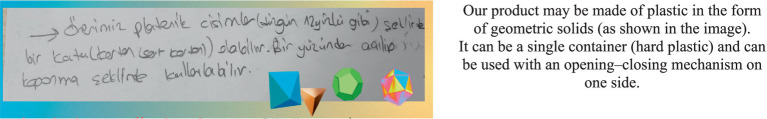
Solution figure for reorganization.

During the implementation of the solution, the process was carried out with determination, and the solution was successfully achieved. In addition, an extra suggestion was added. At this stage, the individual’s perseverance and commitment in applying the chosen solution path were assessed (see Figure 6).

The original version of the student paper is shown in the first image above, and the English translation of the original text is presented alongside it. The original version of the student paper is shown in the first image above, and the English translation of the original text is presented alongside it. In the reorganization stage, it was suggested that a box could be designed in the shape of a Platonic solid (such as a regular dodecahedron) made of cardboard. One of its faces could include a flip-top feature for opening and closing.

#### Idea evaluation

**Figure 7:**
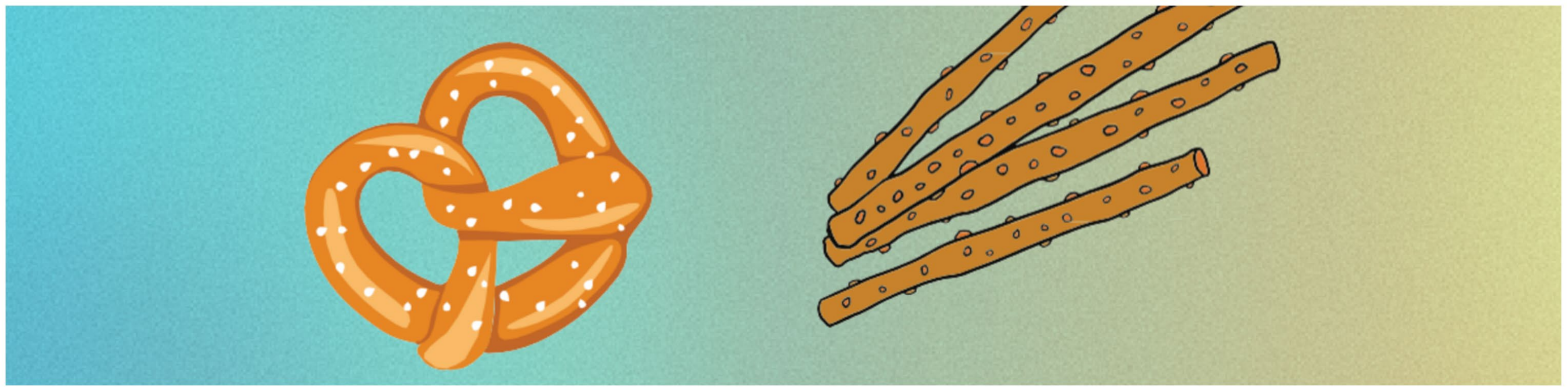
Solution figure for idea evaluation.

The solution path is considered to be original, as every detail of the problem was thoroughly thought out—even down to the type of cracker. Therefore, it can be regarded as an unconventional and distinctive solution (see Figure 7).

In the idea evaluation stage, the design process was meticulously planned and executed.

#### Solution implementation and monitoring

The solution path was successfully implemented. Throughout the process, all possible situations were examined, and the most appropriate solution was reached. The task of increasing the sales of the given product in the problem was successfully accomplished. In the product design, detailed consideration was given to the outer shape of the box, the type of material used, the methods of protection, and the coating material. A potential design was planned, resulting in a distinctive and eye-catching package proposal.

In conclusion, according to Mumford et al.’s (1991) criteria for evaluating the “Ideas in a Box Handout” problem, in the problem construction stage, it was determined that the problem was well understood and possible situations were noted. During the information encoding stage, it was observed that known information was identified and effectively encoded and mentally utilized. In the category search stage, categories for different solution paths were explored, and potential solution categories were recognized. In the category selection stage, the appropriate category was chosen, and the method of solution was determined. In the category combination and reorganization stage, the selected categories were combined. In the idea evaluation stage, all generated ideas were examined, and selections were made among possible alternatives. In the solution implementation and monitoring stage, the solution path was successfully completed, and the results were verified.

## Findings related to the second problem

The analysis results of the responses given by the study group to the creative problem titled “Trick or Treat” were evaluated according to the creative problem-solving criteria proposed by Mumford et al. (1991), as presented below.

(1) Problem Construction

**Figure 8:**
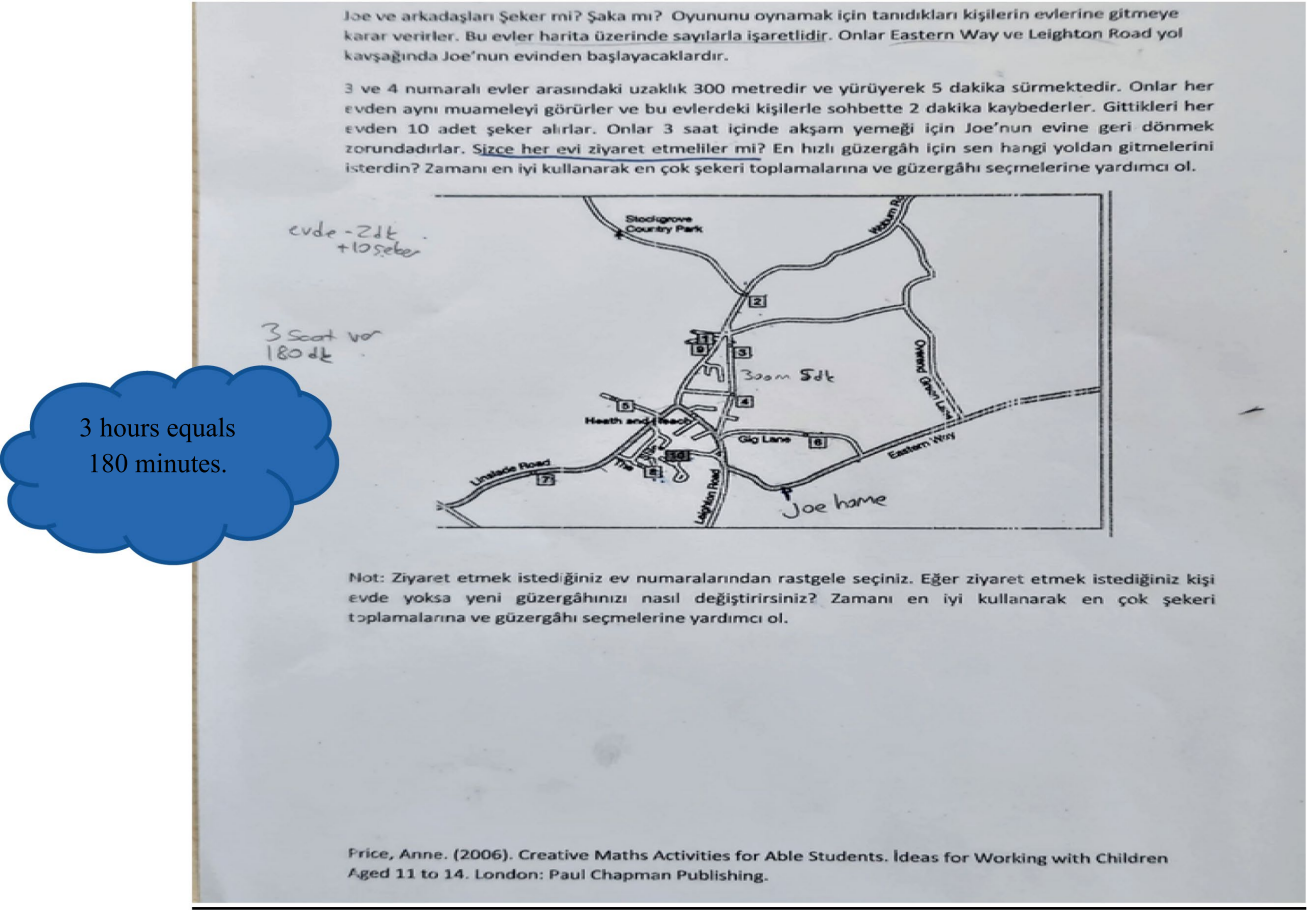
Solution figure for problem construction.

At this stage, the given information in the problem was noted directly on the text. The requirements of the problem were also identified by underlining them. Thus, for the problem construction stage, the given and the required elements of the problem were determined (see Figure 8).

The text in the blue cloud image above is the English translation of the original text. In the problem construction stage, identifying the given and required elements from the problem text is the fundamental step. The required condition was underlined in the problem text. A plan for possible situations was indicated on the visual (e.g., 2 min + 10 candies / 3 h = 180 min).

(2) Information Encoding

**Figure 9:**
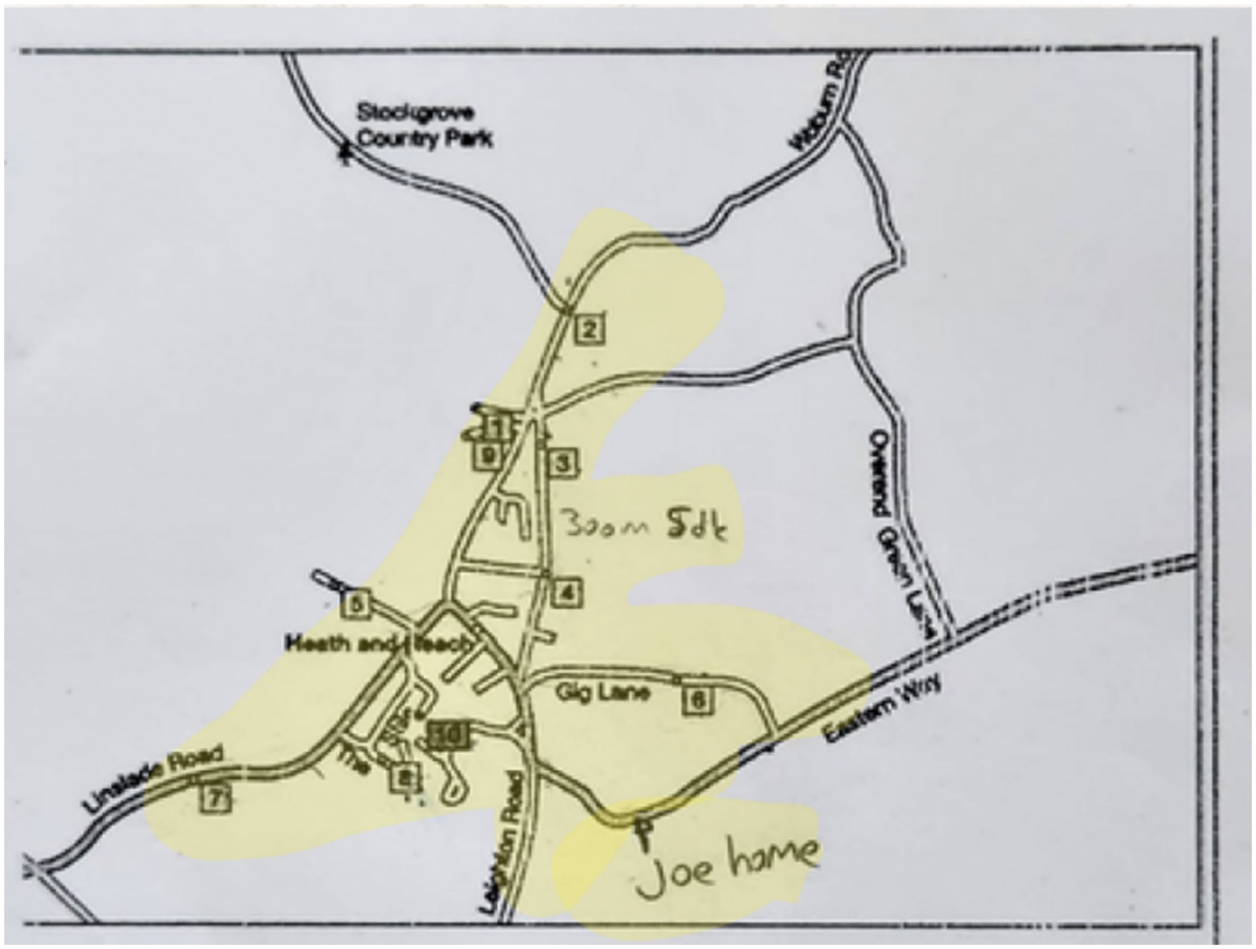
Solution figure for information encoding.

In the information encoding stage, the problem was approached from different perspectives—such as determining the route based on time, maximizing the number of candies collected, and figuring out how to identify in advance whether someone was at home. Various trials were carried out on the map in an attempt to reach a solution. Therefore, the information encoding stage was completed (see Figure 9).

In the visual above, the shaded area indicates the possible routes for collecting a greater number of candies. The distance between houses 3 and 4—300 meters and 5 min—was noted directly on the problem text. Since the problem stated that Joe’s house is located at the intersection of Eastern Way and Leighton, it was approximately marked on the map.

(3) Category Search

**Figure 10:**
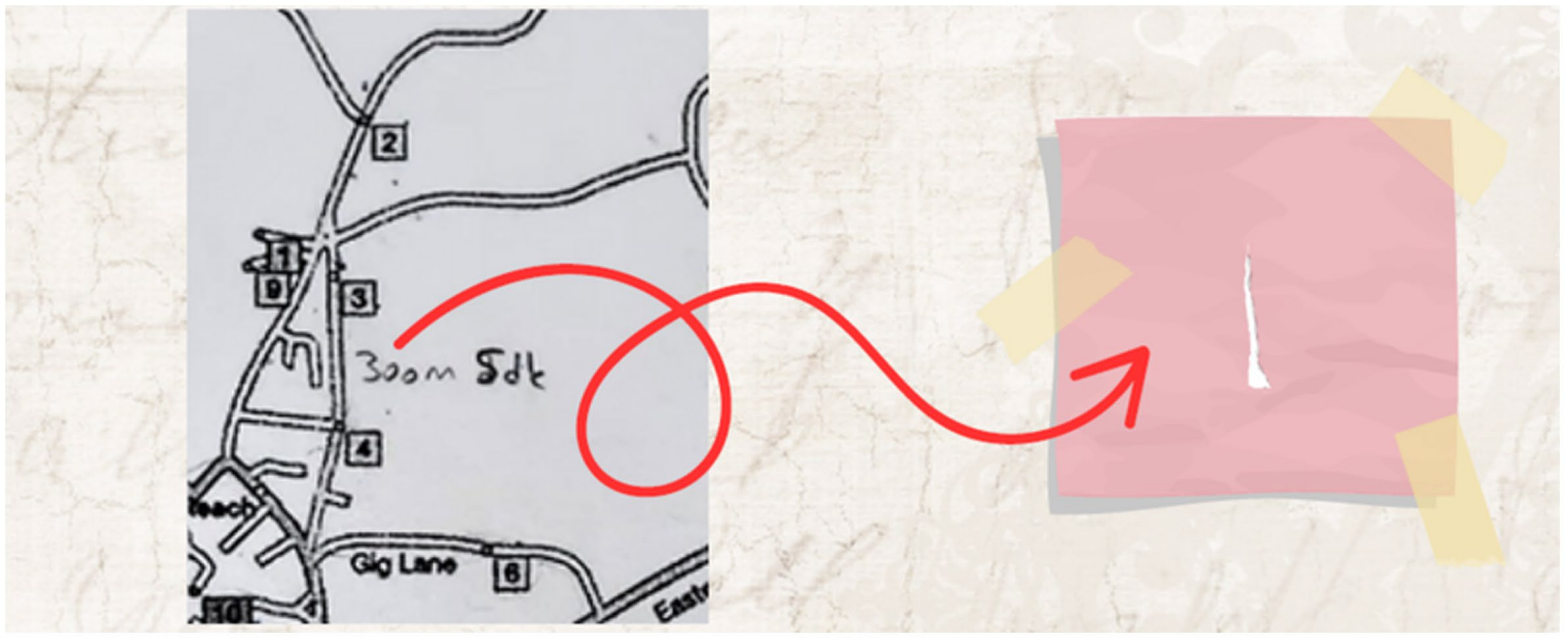
Solution figure for category search.

Within the allotted time for solving the problem, two different solution paths were generated. Using the given route as a reference, distances were measured with a piece of paper, and each possible path was tested individually using this method. At this stage, several alternative solution routes were explored (see Figure 10).

During the category search stage, specific action plans were designed. A piece of paper was used as a measuring tool to plan the routes.

(4) Category Selection

**Figure 11:**
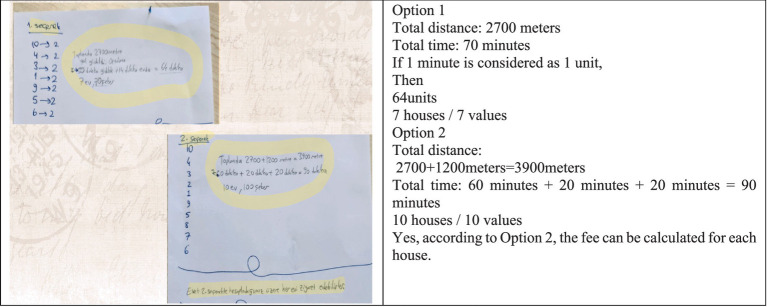
Solution figure for category selection.

Among the two different solution paths generated, the second solution was determined to be the more logical option within the time constraints given in the problem (see Figure 11).

An English translation of the student responses displayed in the image above is included in the adjacent column. In the category selection stage, a relationship was established between time and the number of candies. It was noted that the second option would lead to collecting more candies after visiting each house.

(5) Category Combination

**Figure 12:**
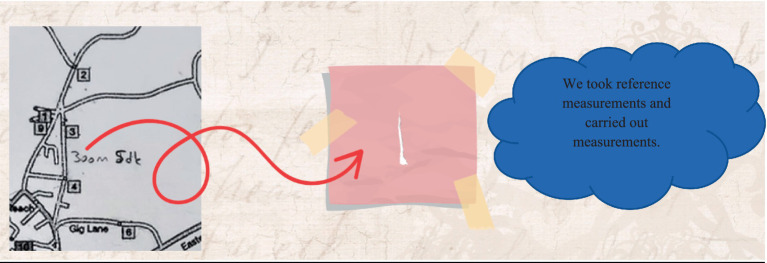
Solution figure for category combination.

While implementing the second solution path, new routes were explored to obtain a more concrete solution. Possible solution paths were tested using a small piece of paper (see Figure 12).

In the category combination stage, it was noted that different measurements were taken and several trials were conducted. The ideas derived from these trials were addressed in the reorganization stage.

(6) Reorganization

It was evident that the participant did not refrain from conducting trials during the implementation of the solution path. By asking various questions that prompted reflection on the solution, the participant demonstrated determination and successfully reached the outcome.

**Figure 13:**
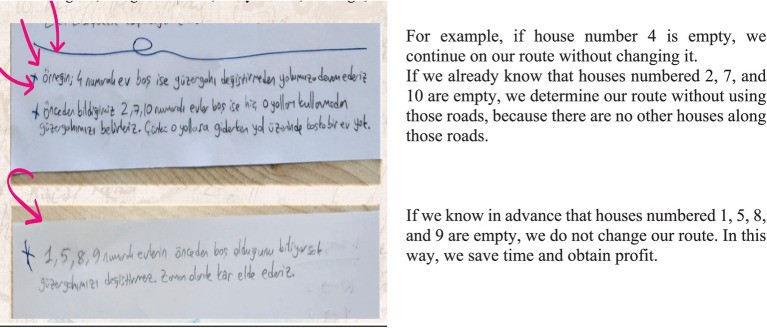
Solution figure for reorganization.

The English translation of the student responses shown in Figure 13 is provided alongside. The examples provided for the reorganization stage are shown in the visual above. The focus was on selecting the route based on whether the houses were occupied or vacant. For example, it was stated that if house number 4 was vacant, the route would continue without any change. Additionally, if houses 2, 7, and 10 were known to be vacant beforehand, those routes would be avoided, as there were no other houses along those paths. Furthermore, if it was already known that houses 1, 2, 8, and 9 were vacant, it was noted that there would be no need to change the route at all, resulting in time savings.

(7) Idea Evaluation

**Figure 14:**
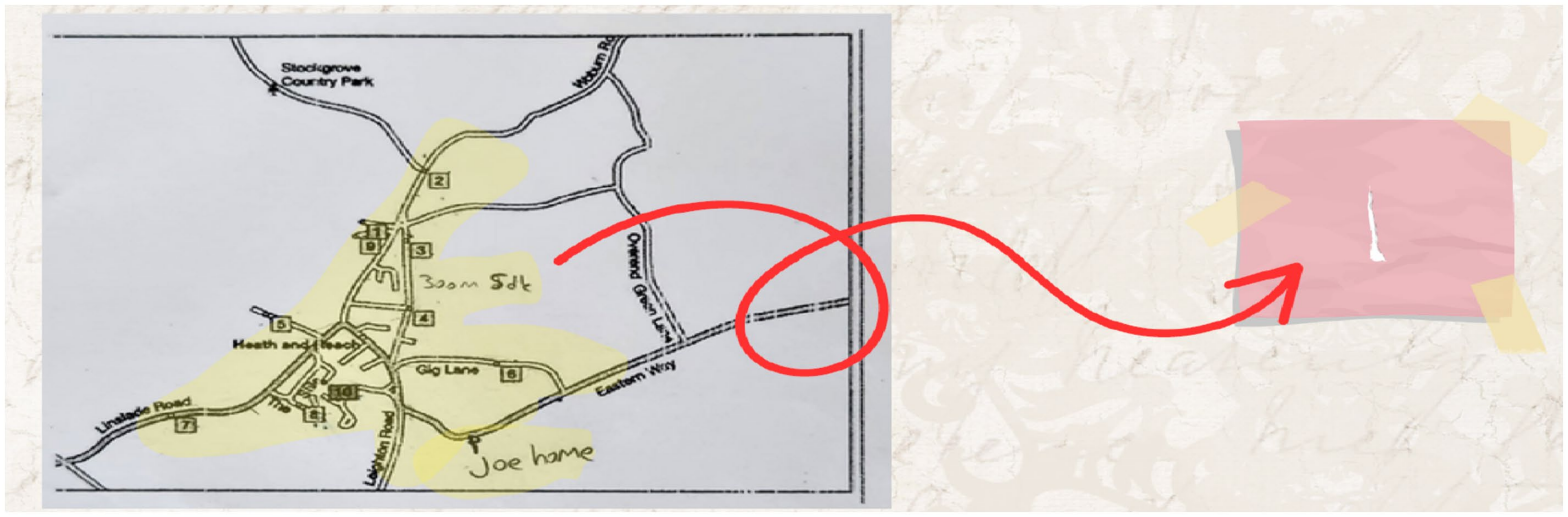
Solution figure for idea evaluation.

During the solution process, no information was available regarding the exact lengths of the routes. Therefore, the only given distance was taken as a reference length, and proportional measurements were made accordingly. An unconventional method of measurement was applied using a piece of paper (see Figure 14).

In the idea evaluation stage, the concept of using a piece of paper for measurement was considered a highly valuable idea. A concrete solution was achieved through proportional reasoning based on this method.

(8) Solution Implementation and Monitoring

After conducting various trials and exploring alternative possibilities, the final solution was successfully reached. From the construction of the problem to its evaluation, the process of collecting candies—by visiting as many houses as possible within the given time—was effectively completed.

As a result, the students carefully examined the locations and distances of the houses on the map. The problem stated that the distance between houses 3 and 4 was 300 meters, which could be covered on foot in 5 min. It was also mentioned that each house was inhabited, that greeting a resident took 2 min, and that each resident rewarded the greeting with 10 candies. Based on these conditions, students were asked to start from house number 10, visit as many houses as they wished, and return to house number 10 within 3 h. They were expected to visit multiple houses, collect as many candies as possible, and return home on time.

### Findings related to the third problem

The findings regarding the sub-problem “How do the study group’s responses to the two creative problems compare with the scoring criteria of the creative thinking test?” are presented below. The responses given by the students for the seven activities of the Torrance Verbal Forms A and B were analyzed under the main theme of Creativity, as outlined below.

The first activity belongs to the “Asking Questions” category, and the analyses of its subcategories and codes are presented (Table 1).

**Table 1:** Subcategories and codes related to the first activity—asking questions category.

Theme: Creativity		
Category	Subcategory	Codes
Asking questions (Verbal A)	Physical qualities of the environment	Sea (2), Lake (2), Puddle (2), Mirror on the ground, Muddy place, Reflection
	Physical movement	Hiding, Slipped, Dropped an item
	Place	Looking at fish, measuring depth
	Emotions	Fear, Facial expression
Asking questions (Verbal B)	Costume	Shoes
	Animal	Type of bird, Harmful, Parrot talking
	Place	Rear window, Location, Country, House
	Physical movements	Running away (2), Man hits woman, Holding the animal, Man standing bent, Blocking, Talking, Looking in opposite directions
	Emotions	Anger, Argument, Problem, Fear
	Profession	Job (2)
	Symbols	Meaning (5), Cracks (2)

The table above presents the qualitative analysis of the responses provided by the students in the “Asking Questions” section of the Torrance Verbal A–B Forms’ first activity. In this activity, students were asked to generate questions and make predictions based on a given visual stimulus. The analysis revealed that students’ questions were primarily focused on physical characteristics, emotions, places, movements, social relations, and symbols.

According to the findings from the Verbal Form A, students tended to focus more on the physical qualities of the environment (e.g., sea, lake, puddle, muddy area). Within the physical movements subcategory (e.g., hiding, slipping, dropping an item), they demonstrated causal reasoning by establishing cause–and–effect relationships. Their questions were also oriented toward spatial and goal-directed thinking (e.g., looking at fish, measuring depth). In the emotions subcategory (e.g., fear, facial expression), students made predictions related to the inner world of the characters.

According to the findings from Verbal Form B, students demonstrated a broader range of diversity in their responses, covering categories such as costumes, animals, places, professions, and symbols. Within the question-asking subcategory, students explored social relationships and conflicts (e.g., “the man hits the woman,” “argument,” “problem”). Emotions such as anger and fear were explicitly expressed in the questions. Furthermore, the production of intensive questions related to symbols (e.g., meaning, cracks) indicates students’ ability to abstract the visual stimulus and attribute meaning to it. In terms of fluency, students’ ability to generate numerous questions about different objects, events, emotions, and symbols suggests a high level of fluency. The greater diversity of perspectives observed in Verbal Form B can be interpreted as evidence of the effectiveness of the creative problem-solving instruction provided during the study. Regarding flexibility, the variation of questions across both concrete (e.g., sea, lake, bird species) and abstract (e.g., meaning, emotions, argument) dimensions reflects students’ flexible thinking skills. The originality dimension was particularly evident in questions related to symbols and social relationships, which went beyond ordinary observation and demonstrated students’ capacity for original and unique thinking.

The findings reveal that in the “Ask and Guess” activity of the Verbal Form B, students not only focused on directly observable elements but also questioned emotional, social, and symbolic dimensions. When evaluated in terms of Torrance’s creativity dimensions, this indicates that students demonstrated remarkable performance in flexibility and originality. Consequently, it can be concluded that the creative problem-solving activities had a positive impact on the question-asking category.

The second activity, “Guessing Causes,” involves analyzing the subcategories and codes presented (see Table 2).

**Table 2:** Subcategories and codes related to the second activity—guessing causes category.

Theme: Creativity		
Category	Subcategory	Codes
Guessing Causes (Verbal A)	Object	Dropped an item (4), Mirror (2), Antique piece, Costume
	Physical activity	Watching fish (2), Seeing reflection (2), Fishing (2), Looking at water (4), Washing clothes, Washing face, Smelling water, Drinking water, Running away, Falling down
	Magic	Wizard
	Emotion	Inspiration
	Place	Pond
Guessing Causes (Verbal B)	Physical action	Running away (4), Stealing the animal (3), Man wants to leave, Woman says do not go, They will sell the animal, They will protect the animal
	Natural disaster	Earthquake (2), Mouse (2)
	Emotions	Fear (4), Thief, Fight (4)
	Symbols	Figurine

In the Physical Action subcategory, most participants attempted to explain events through concrete actions. Among the codes were behavior-based predictions such as “running away” (4), “stealing the animal” (3), “the man wants to leave,” “the woman says do not go,” “they will sell the animal,” and “they will protect the animal.” This finding indicates that students generally constructed observable and realistic scenarios. The diversity of actions reflects a high level of fluency, though it largely remained tied to everyday life experiences. In the Natural Disaster subcategory, some responses referred to environmental causes, such as “earthquake (2)” and “mouse (2).” This shows that students tended to think about environmental phenomena within cause-and-effect relationships. In particular, the “earthquake” code points to a non-visible but mentally conceivable event, reflecting abstract thinking skills. In the Emotions subcategory, participants’ responses contained emotional elements such as “fear (4), thief, fight (4).” Here, it is evident that students explained events not only through physical causes but also through psychological and social dynamics. The frequent occurrence of “fear” and “fight” suggests that issues like safety, threat, and conflict, commonly encountered in their experiences, were reflected in their cognitive processes. Finally, in the Symbols subcategory, although limited, the code “figurine” emerged as a symbolic element, indicating that some students tended toward abstract or metaphorical thinking. However, the level of symbolic reasoning was found to be more limited compared to the other categories.

In general, when both forms (Torrance Verbal A and B) are considered, participants generated a large number and variety of causes, indicating a high level of fluency. In terms of flexibility, the responses were distributed across various categories, including physical action, natural disaster, emotion, and symbols, demonstrating the presence of flexible thinking skills. Regarding originality, most responses were ordinary and connected to daily life experiences, showing that originality remained somewhat limited in this activity. However, a few distinctive ideas, such as the figurine, can still be considered as examples of original thought. In the second activity, students primarily focused on realistic and concrete causes, while emotional explanations also played a significant role. However, the limited number of symbolic and original responses suggests a dimension that needs further development in terms of creativity. As a result, it can be concluded that the creative problem-solving course contributed positively to fluency and flexibility in the “guessing causes” process, but there remains a need to enhance originality through further activities.

The third activity, “Guessing Consequences,” involves analyzing the subcategories and codes presented (see Table 3).

**Table 3:** Subcategories and codes related to the third activity—Guessing consequences category.

Theme: Creativity		
Category	Subcategory	Codes
Guessing Consequences (Verbal A)	Emotions	Satisfaction, Regret, Madness
	Magic	Wizard
	Environment	Mirror shop
	Physical action	Falling (2), Hat dropped (2), Washed face, Fishing (2), Found what was dropped, Found a sea creature, Might ask for help
Guessing Consequences (Verbal B)	Place	The house collapsed, an animal entered the house
	Physical movement	They are kidnapping the animal (4), They are rescuing the animal (3), They sold the animal (2), They are running away (2), They are moving, They were caught by the police, They handed the animal over to the authorities
	Emotions	Fear, Fight, Argument
	Natural disaster	Earthquakes, they lost their homes, lost their lives, lost their animals

In the Verbal Form A—Guessing Consequences category, the emotions subcategory included codes such as satisfaction, regret, and madness, indicating that students associated events not only with physical causes but also with emotional outcomes. This can be interpreted as a reflection of emotional intelligence and social awareness within the creative thinking process. In the magic subcategory, the code “wizard” demonstrates a tendency toward fantastical and imaginative thinking, which represents an important finding that supports originality. The code “mirror shop” in the environment subcategory suggests that students went beyond ordinary thinking patterns and generated creative spatial settings. In the physical action subcategory, responses such as falling (2), hat dropped (2), washing face, fishing (2), finding what was dropped, finding a sea creature, might ask for help, show that students largely focused on observable, everyday life-related consequences. These codes indicate a high level of fluency, though the degree of originality remained limited.

In the Verbal Form B—Guessing Consequences category, the place subcategory included codes such as “the house collapsed” and “an animal entered the house,” showing that events were predicted based on concrete spatial changes. In the physical movement subcategory, most students focused on animal-centered outcomes, such as “they are kidnapping the animal (4), they are rescuing the animal (3), they sold the animal (2), they are running away (2), they are moving, they were caught by the police, they handed the animal over to the authorities.” Both negative scenarios (kidnapping, selling) and positive ones (rescuing, handing over) were presented, illustrating a strong dimension of flexibility. In the emotions subcategory, codes such as fear, fight, and argument demonstrate that social relationships and emotional consequences were reflected in students’ event constructions. These types of emotional explanations reveal the psychosocial dimension of creative thinking. In the natural disaster subcategory, codes such as earthquake, losing their homes, losing their lives, and losing their animals indicate that students associated events with dramatic and catastrophic scenarios. These responses show that students were able to abstract situations and consider a wide range of possibilities in their reasoning.

In the Guessing Consequences category, for the fluency dimension, it can be stated that in both Verbal Forms A and B, students produced a large number of predictions, demonstrating their ability to generate different outcomes quickly. In terms of flexibility, responses were distributed across various categories—ranging from physical actions to emotions, natural disasters, and spatial changes—indicating a strong level of cognitive flexibility. For the originality dimension, while some responses such as “wizard,” “mirror shop,” and “they handed the animal over to the authorities” reflected elements of originality, most predictions were based on ordinary, real-life situations. Students’ predictions of outcomes were generally realistic, concrete, and emotionally grounded. However, originality was represented by only a limited number of examples. Overall, this suggests that within the creative thinking process, there is a need to further develop originality and symbolic thinking. Consequently, it can be concluded that creative problem-solving activities remained limited in supporting the originality dimension.

The fourth activity, “Product Improvement,” includes the analysis of subcategories and codes presented (see Table 4).

**Table 4:** Subcategories and codes related to the fourth activity—product improvement category.

Theme: Creativity		
Categories	Subcategories	Codes
Product Improvement (Verbal A)	Movement	Attaching wire to hose (2), Spraying, Adding wheels, Making it sit, Making it walk, Drinking water
	Structural modification	Color, light, eyes
	Technology integration	Artificial intelligence integration (2), Tracking device, Adding buttons to feet
	Humanization	Adding clothes (2), speaking English (2), giving commands (2), singing, and Imitating
Product Improvement (Verbal B)	Humanization	Dressing it in colorful clothes (2), putting on a wig, making it talk, making it imitate, singing a lullaby, wearing a sports team uniform
	Additions	Sound device (3), Changing into different colors (2), Flying with a propeller, Adding a zipper on the belly, Making it a bag shape, Increasing height, Adding an alarm clock
	Movement	Making it dance (2), Dancing like a monkey, Moving its tail, Making a chewing sound when seeing a banana, Climbing a tree, Jumping
	Technology	Adding an AI device (2), adding light to its eyes (night lamp), singing children’s songs, adding a GPS tracking device, placing a phone inside, and installing a screen on its belly

In the Verbal Form A—Product Improvement category, the movement subcategory included responses such as “attaching wire to the hose (2), spraying, adding wheels, making it sit, making it walk, drinking water,” indicating that students attempted to add functional motion features to the product. This reflects a practical and utilitarian mindset. In the structural modification subcategory, expressions such as “color, light, eyes” represent simple yet creative visual enhancements, demonstrating a focus on the aesthetic dimension of the product. In the technology integration subcategory, responses such as “artificial intelligence integration (2), tracking device, adding buttons to the feet” show that students aimed to introduce a technological dimension to the product. This approach reveals that students’ awareness of contemporary technology was reflected in their thinking processes. The humanization subcategory, including responses like “adding clothes (2), speaking English (2), giving commands (2), singing, imitating,” indicates a tendency to equip the product with human-like qualities. Humanization activates imagination and enhances the potential for originality.

In the Verbal Form B—Product Improvement category, the humanization subcategory included responses such as “dressing it in colorful clothes (2), putting on a wig, making it talk, making it imitate, singing a lullaby, dressing it in a sports team uniform,” suggesting that the product was transformed into a playful, entertaining, and human-like entity. This approach shows that students were capable of creative analogical thinking. The additions subcategory, including responses such as “sound device (3), change into different colors (2), make it fly with a propeller, add a zipper on its belly, make it bag-shaped, increase its height, add an alarm clock,” demonstrates that students offered a diverse range of ideas to enhance the product’s functionality, reflecting a high level of fluency. In the movement subcategory, responses such as “make it dance (2), dance like a monkey, move its tail, make a chewing sound when seeing a banana, climb a tree, jump” show that the product was enriched with movement-based capabilities. These responses indicate that students generated imaginative and playful creative ideas. Finally, in the technology subcategory, responses such as “add an AI device (2), add light to its eyes (night lamp), make it sing children’s songs, add a CPS tracking device, place a phone inside, install a screen on its belly” reveal that the product was equipped with advanced technological features. This category stands out in terms of originality, showing that students were able to creatively incorporate contemporary technological developments into their conceptualizations.

In terms of fluency, students in both Verbal Form A and Verbal Form B produced a large number of ideas. In particular, the “additions” and “movement” categories indicate that the level of fluency was quite high. This outcome can be attributed to the fact that the participating students were graduates with teaching experience, which likely enhanced their capacity to generate a variety of ideas within a limited timeframe. Regarding flexibility, the ideas were distributed across different domains such as movement, aesthetics, technology, and humanization, demonstrating that students were able to develop multiple perspectives and approach the problem from diverse angles.

As for originality, some responses—such as “artificial intelligence integration, bag-shaped design, adding a zipper to the belly, making it fly with a propeller”—were highly original, while others—such as “adding clothes” or “making it talk”—remained relatively ordinary and conventional. In the fourth activity, it can be concluded that students produced both functional and aesthetic solutions during the product development process. Technological and humanizing elements were particularly prominent as areas where creativity was most evident. However, for most students, originality remained confined to common, familiar ideas, gaining a more creative dimension only through a few responses (e.g., adding a zipper, making it fly with a propeller).

Overall, it can be stated that students experienced challenges in moving beyond conventional thinking and struggled to reach truly original ideas during the creative problem-solving activities.

The fifth activity, “Unusual Uses,” involves analyzing the subcategories and codes presented (see Table 5).

**Table 5:** Subcategories and codes related to the fifth activity—unusual uses category.

Theme: Creativity		
Categories	Subcategories	Codes
Unusual uses (Verbal A)	Household items	Blanket, Christmas tree, Laundry basket, Storage container, Doormat, Feeding bowl
	Furniture	Coffee table, Bookshelf
	Games	Basketball court, Goalpost, Hoop, Toy, Matching cards
	Education	Protractor, Material, Cube
Unusual uses (Verbal B)	Household items	Coffee table, Feeding bowl, Table, Spice jar, Storage container, Cup, Lamp, Chandelier, Disco ball
	Toys	Cat toy, Ping-pong table, Scarecrow, Bowling pin, Drum, Goalpost, Goblet drum, Musical instrument
	Education	Pencil holder, Piggy bank
	Animal shelter	Cat house, Bird feeder

In the Verbal Form A—Unusual Uses category, under the Household Items subcategory, ideas such as “blanket, Christmas tree, laundry basket, storage container, mat, pet bowl” indicate that students adapted everyday objects to new contexts. This category emphasizes practical and functional thinking.

In the Furniture subcategory, the responses “table, bookshelf” demonstrate creative thinking focused on transforming the object into a piece of furniture.

Within the Games subcategory, ideas such as “basketball court, goalpost, hoop, toy, matching cards” reveal that students were able to transfer the object into a play and entertainment context. This can be seen as a strong area reflecting children’s imagination.

In the Education subcategory, answers like “protractor, cube, material” show that the object could be transformed into an educational tool, pointing to abstraction and use in academic contexts.

In the Verbal Form B—Unusual Uses category, under the Household Items subcategory, answers such as “table, pet bowl, desk, spice jar, storage container, glass, lamp, chandelier, disco ball” demonstrate that the product was considered for a wide range of domestic functions. Especially examples like “chandelier” and “disco ball” indicate more original ideas that go beyond ordinary use. In the Toys subcategory, answers such as “cat toy, ping-pong table, scarecrow, bowling pin, drum, goalpost, darbuka, musical instrument” show that the product was associated with play and music, leading to both creative and entertaining uses.

In the Education subcategory, ideas like “pencil holder, money box” indicate that the object was repurposed as an educational or organizational tool. Finally, in the Animal Shelter subcategory, responses such as “cat house, bird feeder” demonstrate that the product was redesigned for animal-related purposes, reflecting students’ empathy and environmental awareness.

As a result, in the fluency dimension, students generated a large number of different usage ideas. Numerous alternatives were suggested for both household and play/entertainment purposes. In the flexibility dimension, ideas spread across diverse domains such as household items, furniture, games, education, and animal shelters, indicating a strong level of flexibility. In the originality dimension, while some responses were rather ordinary (e.g., table, desk, storage container), others such as “chandelier, disco ball, bird feeder, darbuka” enhanced the level of originality.

In the Unusual Uses activity, students produced functional, entertaining, and original ideas by taking objects beyond their everyday functions. Suggestions related to games, music, and animal shelters particularly highlight the strong aspect of creativity. However, the repetitive and ordinary nature of some responses indicates that not all students utilized originality equally.

In conclusion, within the Unusual Uses category of creative problem-solving activities, it can be stated that students differed in their ability to produce original ideas.

The sixth activity belongs to the “Unusual Questions” category, and the analyses of its subcategories and codes are presented (see Table 6).

**Table 6:** Subcategories and codes for the sixth activity (unusual questions category).

Theme: Creativity		
Category	Subcategory	Codes
Unusual Questions (Verbal A)	Simple	Color, Forming an object, Game
	Complex	Durability (2), Raw material, Prism, Liquid transport
Unusual Questions (Verbal B)	Simple	Combination, Material, House, Durability, Piggy bank
	Complex	Recycling, How many triangles can we line up side by side?, Do height and width affect sound?, How to make a hole?, How many can we knock down in one hit?, Making a ship, Tiling, Three-dimensional sculpture, Which notes are produced when hit?, Orchestra, Disco ball

In the Verbal Form A—Unusual Questions category, within the Simple Questions subcategory, questions such as “color, forming an object, game” reflect students’ more superficial and concrete curiosity about the object. These questions are based on basic observations and contain functional curiosity rather than creativity. In the Complex Questions subcategory, questions like “durability (2), raw material, prism, liquid transport” reveal a focus on the technical and structural properties of the object. This indicates that students questioned the object not only in terms of its usage but also its scientific and functional aspects.

In the Verbal Form B—Unusual Questions category, within the Simple Questions subcategory, questions such as “combining, material, house, durability, money box” show that students related the product to everyday life contexts. Here, the questions remained at a more practical and functional level of thinking. In the Complex Questions subcategory, questions such as “recycling, how many triangles can be placed side by side, do height and width affect sound, how to make a hole, making a ship, tiling, three-dimensional sculpture, what notes are produced when struck, orchestra, disco ball” are particularly noteworthy. These questions demonstrate that students examined the object from physical, artistic, and scientific perspectives. Especially questions like “orchestra” and “disco ball” reveal the emergence of aesthetic and artistic thinking.

As a result, it can be stated that in the fluency dimension, students generated many simple and complex questions, indicating that fluency was strong. In the flexibility dimension, the questions were distributed across a wide range of areas: physical properties (durability, raw material), mathematical concepts (prism, triangle), artistic contexts (notes, orchestra, sculpture), and functional uses (house, money box). This suggests a high level of flexibility. In the originality dimension, questions such as “orchestra, disco ball, three-dimensional sculpture” were highly original, while questions like “house, money box, durability” were more ordinary. In other words, originality appeared strongly in some students, whereas others remained at a more routine level of thinking. In the Unusual Questions activity, students demonstrated the ability to question objects from multiple perspectives. While simple questions were mostly focused on daily life and practical use, complex questions shifted toward scientific, mathematical, and artistic dimensions. It can be said that in the Unusual Questions category, students showed development in fluency, flexibility, and originality in their approach to creative problems.

The seventh activity belongs to the “Suppose That” category, and the analyses of its subcategories and codes are presented (see Table 7).

**Table 7:** Subcategories and codes for the seventh activity (Suppose that category).

Theme: Creativity		
Categories	Subcategories	Codes
Just Suppose (Verbal A)	Movement	Climbing (2), Setting up a swing (2), Coming down, Playing games, Traveling with clouds, Standing still, Balance
	Weather conditions	Rainy (2), Sunny, Weather events, Rain/snow falling in the same place, Shadow
Just Suppose (Verbal B)	Movement	People walking on the roads, no greetings, we cannot use phones
	Weather conditions	Ecosystem, Atmosphere
	Emotions	Unprejudiced (2), Joyless (3), Emotionless, Emotion state unclear
	Additions	Name tags attached to feet (2), Lights attached to feet, Photos attached to feet
	Technology	GPS device attached to shoes, Fog vision camera developed, Greenhouses established with new technology
	Profession	Profession recognized by shoes (2). All professions wear the same shoes. They do not do their jobs well
	Economy	Understandably, the Textile trade would decrease

In the Verbal Form A—Suppose That category, under the Movement subcategory, codes such as “climbing (2), building a swing (2), descending, playing games, walking among clouds, standing still, balance” show that students focused on the concept of movement. Some responses are realistic (climbing, balance), while others are imaginative (walking among clouds), revealing the combined use of functional and creative thinking. In the Weather Conditions subcategory, expressions such as “rainy (2), sunny, weather events, rain/snow falling in the same place, shadow” indicate that students incorporated weather phenomena into their story constructions. Especially the idea of “rain/snow falling in the same place” reflects an unusual and creative observation of nature.

In the Verbal Form B—Suppose That category, under the Movement subcategory, statements such as “People walk on the roads, there is no greeting, we cannot use phones” show that students considered daily life under different assumptions. Here, there is a creative questioning of social behavior. In the Weather Conditions subcategory, ideas such as “ecosystem” and “atmosphere” are related to nature and the environment, suggesting that students imagined with ecological awareness. These responses reflect the environmental dimension of creative thinking. In the Emotions subcategory, answers such as “unbiased (2), joyless (3), emotionless, emotional state unclear” show that students explored human behavior through an emotional lens, indicating that social–emotional awareness was integrated into their creative thinking. In the Additional Features subcategory, expressions like “name tags on feet (2), lights attached to feet, photos attached to feet” demonstrate that objects were imagined beyond their ordinary functions, representing a flexible and innovative perspective. Finally, in the Technology subcategory, ideas such as “CPRS devices attached to shoes, fog-vision camera developed, greenhouses established with new technology” show that technological developments were reflected in creative thought.

It is observed that students developed innovative ideas connected to contemporary life. In the Profession subcategory, statements such as “It would be understood from the shoes (2), all professions would wear the same shoes, they would not do their jobs well” reflect an inclination to question social roles, demonstrating creative thinking about social order. In the Economy subcategory, expressions like “It would be understood, textile trade would decrease” show that students were able to incorporate the economic dimension into their imaginative scenarios—an unusual and original perspective in terms of creativity.

In the fluency dimension, students generated a wide variety of assumptions in both Verbal Form A and Verbal Form B, indicating strong fluency. In the flexibility dimension, expressions spanned across diverse domains such as movement, weather conditions, emotions, technology, profession, and economy, revealing a high level of flexibility. In the originality dimension, responses such as “walking among clouds, rain/snow falling in the same place, attaching photos to feet, greenhouses built with new technology, textile trade would decrease” were particularly original and non-routine ideas.

In conclusion, in the “Suppose That” activity, students produced both realistic and imaginative scenarios, integrating social life, technology, ecology, and economy into their creative thinking. This demonstrates that students were able to imagine not only concrete situations but also abstract and critical dimensions. It can be stated that creative problem-solving activities effectively foster imagination and the emergence of originality.

## Conclusion

This study examined the effect of the Creative Problem Solving (CPS) model on the creative thinking skills of graduate students in mathematics teaching. The findings indicate that CPS-based instructional processes led to significant improvements in the dimensions of fluency, flexibility, and originality. By the end of the process, students not only generated more ideas but also enhanced their ability to evaluate problems from multiple perspectives, develop alternative strategies, and produce original solutions. This study concludes that the CPS model is an effective instructional approach for enhancing students’ creative thinking skills—especially fluency and flexibility—in mathematics education. Although originality showed more limited development, the CPS process supported students in generating more ideas, approaching problems from multiple perspectives, and constructing more meaningful connections between concepts.

Data obtained from the Torrance Verbal Forms A and B revealed that, compared to the pre-application phase, students provided more diverse, detailed, and symbolic responses in the post-test. In terms of fluency, students generated a greater number of ideas, with notable increases in productivity particularly evident in the “Product Development” and “Unusual Uses” activities. Regarding flexibility, it was observed that students expanded their focus beyond physical or concrete explanations to include emotional and abstract dimensions. In the “Predict the Causes” and “Predict the Consequences” activities, students demonstrated diversity across various categories, including emotion, symbol, action, and natural phenomena. The originality dimension emerged strongly in a limited number of students. During the “Product Development” phase, responses such as AI integration, a product in the form of a bag, a toy with a zipper, or a flying toy design reflected a remarkable level of originality.

The TTCT results clearly demonstrated pre- to post-intervention improvement, confirming the positive impact of CPS-based instruction. To further strengthen originality, future instructional activities may incorporate open-ended problem posing, metaphor creation, dramatization, and visual design-based tasks.

The study is limited to five graduate students within a single academic context; therefore, generalizability remains limited. Future research may test the CPS model across different age groups or disciplines, such as science, art, or engineering, to expand its applicability. Longer-term studies and mixed-method designs may also provide deeper insights into the model’s broader impact.

In conclusion, the CPS model presents a promising pedagogical framework for integrating creativity into mathematics education and for fostering students’ higher-order thinking skills.

## Discussion

This study explored the effect of the Creative Problem Solving (CPS) model on the creative thinking skills of graduate students in mathematics education. The findings demonstrated that CPS-based instructional processes contributed meaningfully to the development of fluency, flexibility, and originality—three core components of creative thinking.

The TTCT Verbal Forms A and B results revealed significant increases in students’ ability to generate diverse, detailed, and symbolic responses after the CPS process. Students’ notable improvement in fluency aligns with Scott et al.’s (2004) assertion that CPS enhances productivity and idea generation. Similarly, the flexibility observed in students’ responses—where explanations expanded into emotional, symbolic, and abstract categories—supports studies indicating that CPS fosters multidimensional thinking.

The limited improvement in originality observed in a small number of students reflects findings in previous literature suggesting that originality often requires extended practice and exposure to open-ended, risk-taking environments. Nonetheless, examples such as integrating AI, designing zippered toys, or proposing flying product models indicate promising emerging originality.

Analyses based on Mumford et al.’s (1991) eight CPS stages showed varied student performance across the process. Students performed strongly in problem construction and information encoding, which is consistent with the early cognitive activation described in CPS theory. Group work facilitated idea generation during category search and selection stages, confirming research that collaborative learning enhances divergent thinking. Improvements in category combination and reorganization reflect students’ growing ability to synthesize ideas, a finding supported by Treffinger and Isaksen (2023). Increased ability to justify and evaluate ideas in the later stages aligns with Sriraman’s (2005) view that mathematical creativity involves integration of analytical and intuitive reasoning.

The CPS model thus appears to provide learners with a structured yet flexible framework for engaging in higher-order thinking. Mathematical tasks such as “Trick or Treat” and “Ideas in a Box”—which require real-life application and mathematical modeling—further reinforced creativity development, echoing Leikin and Lev’s (2013) argument that creativity is enhanced through multiple representations and strategy generation.

Overall, the discussion highlights that CPS not only improves creative thinking skills but also enriches mathematical learning experiences by encouraging multidimensional cognitive engagement.

Psychologically, students with stronger creative PS skills typically demonstrate higher cognitive flexibility, which enables them to shift between different ideas, representations, and strategies. They also tend to have greater intrinsic motivation, curiosity, and tolerance for ambiguity—all of which support the exploration of multiple solution paths. These students are more willing to take intellectual risks and generate unconventional responses, which enhances their ability to produce original and diverse ideas. Such characteristics are consistent with theories of creativity that emphasize divergent thinking, openness to experience, and associative fluency. Mathematically, students with higher creative PS capacity can connect mathematical concepts more fluidly and restructure problems in novel ways. Their ability to draw on multiple representations (symbolic, visual, verbal) supports deeper reasoning and facilitates transitions between concrete and abstract ideas. This aligns with the view that mathematical creativity involves flexible strategy use, the ability to generalize patterns, and the capacity to formulate new problem structures. Consequently, these students more easily generate alternative strategies, evaluate solutions critically, and engage in mathematical modeling with greater sophistication.

According to Leasa et al. (2021), students with higher creative thinking skills demonstrate stronger cognitive flexibility, enabling them to generate diverse ideas and shift between conceptual categories more effectively. Their findings show that creativity components such as fluency, flexibility, originality, and elaboration support learners in interpreting problems from multiple perspectives and constructing meaningful associations between ideas. These psychological characteristics also enhance mathematical reasoning, as flexible thinkers are better able to reorganize information, explore alternative strategies, and produce original solutions in problem-solving contexts.

Students with higher creative problem-solving skills tend to demonstrate more advanced psychological and mathematical reasoning capacities. As Batlolona and Diantoro (2023) explain, learners with strong creative thinking skills exhibit higher levels of cognitive flexibility, the ability to form rich mental models, and the capacity to make meaningful connections among ideas. These students are better at reorganizing information, shifting between representations, and generating alternative solutions, all of which support more effective mathematical problem solving. Furthermore, emotionally and cognitively mature students are more capable of constructing mental models that facilitate deeper understanding and higher-order reasoning, leading to superior performance in creative tasks and problem-solving situations.

Although the CPS-based instructional process positively influenced all dimensions of creative thinking, the improvement observed in originality was comparatively more limited than that in fluency and flexibility. This finding can be explained by the nature of originality as a higher-level and more demanding component of creative thinking. Within the CPS framework, fluency and flexibility are primarily fostered through repeated engagement in divergent thinking activities, idea generation, and category shifting, which are emphasized in the early and intermediate stages of the CPS process. These stages encourage the production of multiple ideas and alternative perspectives, making gains in fluency and flexibility more readily observable within a relatively short instructional period. In contrast, originality requires deeper cognitive restructuring, a willingness to take intellectual risks, and the ability to move beyond familiar or socially accepted ideas. Such processes tend to develop more gradually and often require sustained exposure, explicit scaffolding, and learning environments that tolerate ambiguity and failure. From a pedagogical perspective, this finding highlights the importance of teacher professional development programs that focus not only on implementing CPS stages, but also on designing instructional practices that explicitly nurture originality—such as open-ended problem posing, reflective discussion, metaphorical and analogical thinking, and opportunities for students to justify unconventional solutions. For future research, longitudinal and mixed-method studies are recommended to examine how originality evolves over extended CPS interventions and across different educational levels and contexts. Investigating instructional conditions that more effectively support originality may further clarify the long-term potential of CPS-based mathematics instruction in fostering advanced creative thinking skills.

### Recommendations

It is recommended to use the CPS model regularly in mathematics instruction, as the process has led to notable improvements in creativity dimensions such as fluency, flexibility, and originality. To enhance students’ levels of originality, it would be beneficial to incorporate CPS-supportive activities more frequently, such as open-ended problem posing, metaphor creation, gamification, dramatization, and visual design. Since group work increases idea generation during the CPS process, creating collaborative learning environments in mathematics classes is also recommended. Integrating creativity assessment tools such as the TTCT into instructional processes may help monitor students’ creative thinking development more closely.

### Limitations

Since the study was conducted with only five graduate students, the findings cannot be generalized to a larger population and are limited to the context of a qualitative case study. The development in the originality dimension was limited; this may be due to the activities used not sufficiently stimulating this particular aspect of creativity. The research is limited to a single-term implementation; longer-term interventions could enable a broader and more detailed impact analysis.

A limitation of the present study is the absence of baseline data regarding participants’ prior knowledge, previous learning experiences, and motivational characteristics. As initial participant profiles were not systematically collected, it cannot be asserted with certainty that the observed improvements in creative thinking are attributable exclusively to the Creative Problem-Solving (CPS) model. Future research should incorporate baseline assessments of learners’ academic backgrounds and motivational factors to allow for a more robust interpretation of the effects of CPS-based mathematics instruction.

### Suggestions for further research

Examining the effects of the CPS model across different age groups is important for assessing its developmental appropriateness. Implementing CPS activities in connection with other disciplines—such as science, art, and engineering—may reveal the interdisciplinary dimensions of creative thinking. To better understand the limited development observed in the originality dimension, qualitative interviews and process-oriented analyses could be conducted. The CPS model can be tested with larger samples through mixed-methods studies supported by quantitative data, allowing for comparative analyses of its effectiveness. Conducting content analyses of student products (such as designs, drawings, and models) may enable a more detailed assessment of creativity dimensions.

## Data Availability

The original contributions presented in the study are included in the article/supplementary material, further inquiries can be directed to the corresponding author.
